# High-performance cementitious composites containing nanostructured carbon additives made from charred coal fines

**DOI:** 10.1038/s41598-024-59046-y

**Published:** 2024-04-17

**Authors:** Yuan Gao, Viet Hung Pham, Jennifer Weidman, Ki-Joong Kim, Richard E. Spaulding, Congjun Wang, Christopher S. Matranga

**Affiliations:** 1 National Energy Technology Laboratory, 626 Cochran Mill Road, Pittsburgh, PA 15236 USA; 2 NETL Support Contractor, 626 Cochran Mill Road, Pittsburgh, PA 15236 USA

**Keywords:** Civil engineering, Nanoscale materials, Structural materials

## Abstract

Carbon-based nanomaterials, such as carbon nanoplatelets, graphene oxide, and carbon quantum dots, have many possible end-use applications due to their ability to impart unique mechanical, electrical, thermal, and optical properties to cement composites. Despite this potential, these materials are rarely used in the construction industry due to high material costs and limited data on performance and durability. In this study, domestic coal is used to fabricate low-cost carbon nanomaterials that can be used economically in cement formulations. A range of chemical and physical processing approaches are employed to control the size, morphology, and chemical functionalization of the carbon nanomaterial, which improves its miscibility with cement formulations and its impact on mechanical properties and durability. At loadings of 0.01 to 0.07 wt.% of coal-derived carbon nanomaterial, the compressive and flexural strength of cement samples are enhanced by 24% and 23%, respectively, in comparison to neat cement. At loadings of 0.02 to 0.06 wt.%, the compressive and flexural strength of concrete composites increases by 28% and 21%, respectively, in comparison to neat samples. Additionally, the carbon nanomaterial additives studied in this work reduce cement porosity by 36%, permeability by 86%, and chloride penetration depth by 60%. These results illustrate that low-loadings of coal-derived carbon nanomaterial additives can improve the mechanical properties, durability, and corrosion resistance of cement composites.

## Introduction

Cement composites, including cement paste, mortar, and concrete, are some of the most widely used construction materials in the world. However, their quasi-brittle nature, susceptibility to cracking, low toughness, and low tensile strength have been identified as the main characteristics that result in poor durability and high maintenance costs. Traditional methods to enhance the strength and durability of the cement matrix include the incorporation of reinforcement materials (e.g., steel rebars, macro/microfibers), use of chemical mineral admixtures (e.g., silica fume), or reduction of water-to-binder ratio. In the past decade, the use of nanomaterials in cement composites has received considerable attention. Nanomaterials have the potential to control nanoscale cracks, which can prevent their growth and formation of micro-, or even larger-scale, defects^1,2^.

Two-dimensional carbon nanomaterials, such as graphene, have outstanding mechanical properties, including high tensile strength and Young’s modulus^3^, and remarkable thermal and electrical properties^4,5^. Various graphene-related materials have been investigated for their reinforcing effect within a cement matrix, such as graphene oxide (GO), reduced GO (rGO), and graphene nanoplatelets (GNP). GO is highly dispersible in water due to the oxygen functional groups on GO sheets. However, it has been reported that GO tends to cross-link in cement pore solutions due to the presence of alkaline ions (e.g., Ca^2+^, Na^+^, K^+^)^6^. To prevent cross-linking, a polycarboxylate-based superplasticizer (SP) is typically used in combination with ultrasonic vibration to achieve better dispersion^7^. In addition to the application of GO in cement composites, rGO and GNP have also been studied for the reinforcement of cement composites^8–11^. Reduced GO can further enable multifunctional properties in cement composites because of its electrical conductivity^8,9^. GNP is inexpensive and is produced using a more straightforward synthesis method, which could make it suitable for use in construction materials^10,11^.

It is known that hydration, filling, and bonding are the major intrinsic factors influencing the mechanical properties and durability of cement-based materials. Carbon additives have been shown to impact these factors and enhance the properties of cement composites. The abundant functional groups on oxidized carbon nanomaterials, such as GO, can lead to a seeding effect on cement hydration kinetics by providing additional sites for the nucleation and growth of hydration products. Lu et al.^12^ showed that GO rapidly adsorbs Ca^2+^ at early stages of cement mixing and can accelerate the cement dissolution and hydration processes. Xu et al.^13^ also concluded that the Ca-rich environment around GO facilitates the formation of crystalline hydration products in the cement composites, which in turn leads to better mechanical properties than what occurs in amorphous hydration products. Moreover, it was proposed^14,15^ that graphene sheets can interlock the cement matrix together by linking separate hydration products, thereby more efficiently transferring the loading between hydration phases. Zhao et al.^7^ found that the addition of carbon nanomaterials could delay the formation and growth of cracks. Lastly, carbon nanomaterials can fill in the micropores of the cement matrix, resulting in a refined pore structure and, thus, improved durability^16,17^. However, the degree of improvement from pore filling is expected to be limited for very low dosages of carbon additives, and the filling phenomenon will be minimal for ranges around 0.01–0.05 wt.%.

It is reported that graphene-based materials can benefit cement composites in the following aspects: (1) significant reduction of cement consumption and its environmental footprint due to improved mechanical properties; (2) promotion of innovative architectural and structural designs with decreased self-weight; (3) improved durability as a result of enhanced permeability resistance; (4) enablement of self-sensing capability by imparting electrical conductivity; and (5) enhanced fire resistance due to high thermal conductivity^1,18^. Despite the potential advantages of using carbon nanomaterials in cement composites, the upfront cost of the material is believed to be the main barrier for large-scale implementation in the construction industry^19^. Currently, graphene materials used as additives for cement are produced from graphite, which is listed as a critical mineral and is in high demand for the production of Li-ion batteries^20^. In addition, the production yield of graphene nanoflakes from graphite can be low^18,30^. These issues limit the large-scale deployment of graphene-based materials for construction applications. In this work, coal is considered as an alternative source material for graphene to produce carbon additives for high-performance cementitious composites.

Coal is a carbon-rich feedstock and has been used to produce carbon nanomaterials with different techniques, such as chemical oxidation^21^, chemical vapor deposition (CVD)^22^, and flash Joule heating^23^. Low rank coals have poorly aligned aromatic carbon structures, while high rank coals have carbon lattices with more ordered and layered structures. For this reason, select coal types could be candidates for the large-scale production of carbon nanomaterials using simple mechanical exfoliation methods like the ones described in this manuscript. In addition, the abundance and low-cost of coal makes it suitable for the large markets associated with manufacturing carbon-enhanced composites for construction applications.

In this work, three kinds of carbon nanomaterials from coal feedstocks are produced using three different synthesis methods and their properties are assessed for enhanced cementitious composites. The three carbon nanomaterials are carbon quantum dots (C1), non-oxidized carbon nanoflakes (C2), and oxidized carbon nanoflakes (C3). The three coal-derived carbon additives are first incorporated in cement paste samples and tested for their mechanical properties and durability at different dosages. Concrete samples with two different mix designs are also prepared with these coal-based carbon nanomaterials to assess their compressive and flexural strengths. All three coal-derived carbon additives improve mechanical properties by up to 24% in compressive strength and 19% in flexural strength for cement paste samples. In addition, the coal-derived carbon additives substantially improved durability, producing up to a 60% reduction of chloride ion penetration of the cementitious composites. Moreover, these carbon additives demonstrated obvious enhancement (> 20%) for both compressive and flexural strength in concrete samples, illustrating the effectiveness and potential of coal-based carbon additives for construction and infrastructure applications. The hydration products are characterized and discussed to provide insight into the mechanisms leading to these improvements of properties.

## Experimental section

### Synthesis of carbon nanomaterials from charred coal fines

Charred bituminous Blue Gem coal fines (Knox County, Kentucky, ash content < 1.5%) were obtained from Carbon Technology Company (Bristol, Virginia, United States). The charring process was conducted using a mild gasification process where these coal fines were heated at 650–750 °C under an inert atmosphere for 20 min^27^. These coal fines used to produce this char are a metallurgical industry waste product because they are too small for use in most furnaces. They are charred to remove value-added liquids and prepare them for briquetting into large pieces which then can be re-used for metallurgical purposes or utilized as a manufacturing feedstock for carbon nano-materials and composites, such as the ones discussed in this manuscript.

The coal char was used as a feedstock to synthesize carbon nanomaterials by chemical oxidation (C1) and liquid-phase exfoliation (LPE) (C2 and C3). To prepare C1, coal char was first ground using an enclosed shatterbox (SPEXSamplePrep 8530) for 3 min to produce a fine powder (5–50 µm and see Figure S1). Then, 5.0 g of coal char powder was added to a 200 mL mixture of sulfuric acid and nitric acid (3:1 v/v) in a 1-L flask, and the mixture was refluxed under magnetic stirring at 100 °C for 24 h. After natural cooling to room temperature, the mixture was diluted with 1800 mL deionized water and subsequently neutralized with sodium hydroxide solution. Finally, the mixture was purified by tangential-flow ultrafiltration (KrosFlo®KR2i TFF system, Repligen) using a 1.0 kilo Dalton hollow fiber filtration membrane at a pressure of 8.0 psi, and concentration mode with a concentration factor of 20 for five times. The production yield of C1 was approximately 20%.

For the synthesis of C2, coal char was first ground with the shatterbox for 3 min and then wet ball-milled using an attritor (Szegvari Attritor, Union Process) for 2 h into micron-sized coal char particles (1–5 µm and see Figure S2). Next, 5.0 g of this ball-milled coal char powder was added into 500 mL of water, along with 5 mL of superplasticizer (SP) from BASF MasterGlenium, which is conventionally used as a water-reducing agent in concrete material. The solution was then high-shear mixed at 5000 rpm using a high-intensity shear mixer (L5MA Laboratory Mixer, Silverson) for 2 h in an ice bath. The obtained solution was then centrifuged at 1000 rpm for 1 h. The supernatant was collected as C2 solution, and the concentration was determined. The sediment was also collected and reused as the feedstock for another cycle of LPE.

For the synthesis of C3, coal char was first ground with the shatterbox for 3 min and then wet ball-milled using an attritor (Szegvari Attritor, Union Process) for 2 h into micron-sized coal char particles. The ball-milled coal char powder was then chemically oxidized with a mixture of sulfuric and nitric acid at room temperature before LPE. Briefly, 10.0 g of the ball-milled coal char was mixed with 20 mL of sulfuric acid and nitric acid (3:1 v/v) until the coal char was fully wet and the mixture was kept at room temperature for 5 h. After acid washing by dispersing oxidized coal char in 300 mL DI water, mixing for 10 min, and then centrifuging at 5000 rpm for 10 min for five times, the obtained oxidized coal char was dried at 120 °C for 3 h. The LPE was carried out with the oxidized coal char using the same procedure as that for C2. In detail, 5.0 g of oxidized coal char powder was added into 500 mL of water, along with 5 mL of superplasticizer (SP). The solution was high-shear mixed at 5000 rpm for 2 h in an ice bath. The obtained solution was then centrifuged at 1000 rpm for 1 h. The supernatant was collected as C3 solution and the concentration was measured. The sediment was used as the feedstock for another cycle of LPE.

### Cement composite preparation with three carbon nanomaterials at five different dosages

C1, C2, and C3 were incorporated in cement paste and the composite was then characterized. Since SP was added to both C2 and C3 solutions during the synthesis process, the same amount of SP was added to the C1 solution to ensure uniform dispersion of the carbon additives within the cement matrix. Specifically, a solution of coal-based carbon nanomaterials with concentrations of 0.05 to 0.35 wt.% (in aqueous solution) was mixed with dry cement powder without any additional water and mixed and cured for 28 days according to the ASTM 305^24^. The water-to-cement ratio was 0.2. Cylinder samples with the dimension of 1-in. diameter × 2-in. length were prepared for the compression test, porosity/permeability test, and chloride penetration test with the dosages of each carbon nanomaterial in the range of 0.01% to 0.07% (to cement weight). Prism samples with a dimension of 0.8 in. × 0.8 in. × 3.2 in. were also prepared for the three-point bending test. The permeability/porosity tests and chloride penetration tests were performed with the same set of samples as those used for the compression test. Commercial GO from Graphenea, Inc. was also used as an additive to prepare cement paste samples at the dosage of 0.025 wt.% and 0.05 wt.% for performance comparison with coal-derived additives. Concrete samples with two different mix designs were prepared in cylinders and prisms with the mix ratio shown in Table 1. Mix 1 only contains water, sand and coarse aggregate, which simulates a normal-strength concrete with the compressive strength less than 6000 psi. Mix 2 has a lower water to cement ratio at 0.32, and is absent of coarse aggregate. 5 wt.% of silica fume is added to improve the compressive strength, and it simulates a high-strength concrete with the compressive strength more than 6000 psi. The three carbon nanomaterials were added at the dosage of 0.02 wt.%, 0.04 wt.%, and 0.06 wt.%. Concrete prism samples are in the dimension of 1.57 in. × 1.57 in. × 6.3 in., and concrete cylinder samples are in the dimension of 2.5 in. × 5 in.

**Table 1:** Mix design for concrete samples (weight ratio to cement).

	Mix 1	Mix 2
Water to cement	0.4	0.32
Sand to cement	1.6	1
Coarse aggregate to cement	2.1	0
Fly ash to cement	0	0.2
Silica fume to cement	0	0.05

### Characterization and measurements

XRD measurements were carried out using a PANalytical X’pert pro X-ray diffractometer (XRD) with Cu Kα radiation (λ = 1.5418 Å) with a step size of 0.017° and 200 s/step in the 2θ range from 10° to 70°. The XRD was operated at 45 kV and 40 mA. X-ray Photoelectron spectra (XPS) of the materials were obtained with a PHI 5600ci spectrometer equipped with a hemispherical electron analyzer and a monochromatic Al Kα (1486.6 eV) radiation source. The pass energy of the analyzer was 55 eV. Scanning electron micrographs and energy dispersive X-ray spectra (SEM/EDS) were acquired with a FEI Quanta 600F microscope operated at 10–20 kV. TEM images were taken using a JEOL JEM2100F operated at an accelerating voltage of 200 kV.

For mechanical property testing of cementitious composites, compression tests were performed on cylinder samples according to ASTM C39^25^. Three-point bending tests were performed on prism samples according to ASTM C348^26^. At least three samples were tested for each batch and the average values were taken as the strength of the cement composites.

For porosity and permeability testing, cylinder samples were dried in a desiccator until the mass of samples stabilized. The porosity was tested using a TEMCO Helium Porosimeter HP-401. After porosity tests, each cylinder sample was tested for permeability in a TEMCO Pulse-Decay Permeameter, which sets a pore pressure throughout the sample, sends a differential pulse through the entire sample, and measures travel time to calculate permeability.

The modified chloride penetration test was conducted on cement cylinders according to AASHTO T259, where the top face of the sample was exposed to 3% sodium chloride solution, and the bottom face was exposed to 50% relative humidity of air. After exposure for 30 days, the sample was split into halves vertically, and the split surface was sprayed with silver nitrate. The chloride ion’s penetrated depth was measured for each sample, and the average value was taken from each batch.

The microstructure of the cement composites was analyzed using differential scanning calorimetry (DSC), thermogravimetry analysis (TGA), and XRD. The simultaneous DSC-TGA measurements were conducted using a Mettler Toledo TGA/DSC 3+. The tests were performed from 35 to 1000 °C with a ramp rate of 10 °C /min under the environment of nitrogen gas. TGA was also utilized to examine the mineral matter content of the coal char under the environment of air gas. The XRD pattern was measured with the cement samples using the same condition mentioned above.

## Results and discussion

### Characterization of coal char feedstock

**Figure 1:**
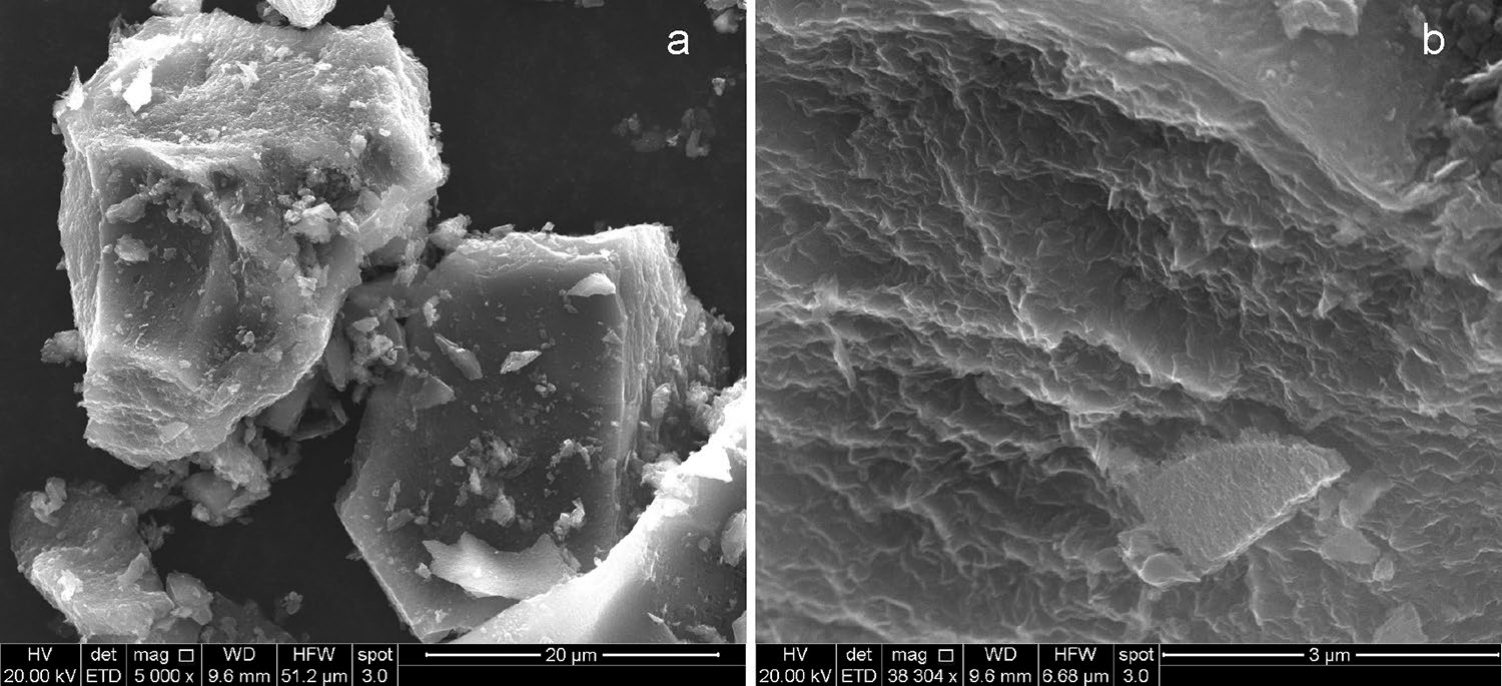
SEM images of Blue Gem coal char precursor used for the synthesis of carbon nanomaterials. Scale bar: (**a**) 20 µm, (**b**) 3 µm.

**Figure 2:**
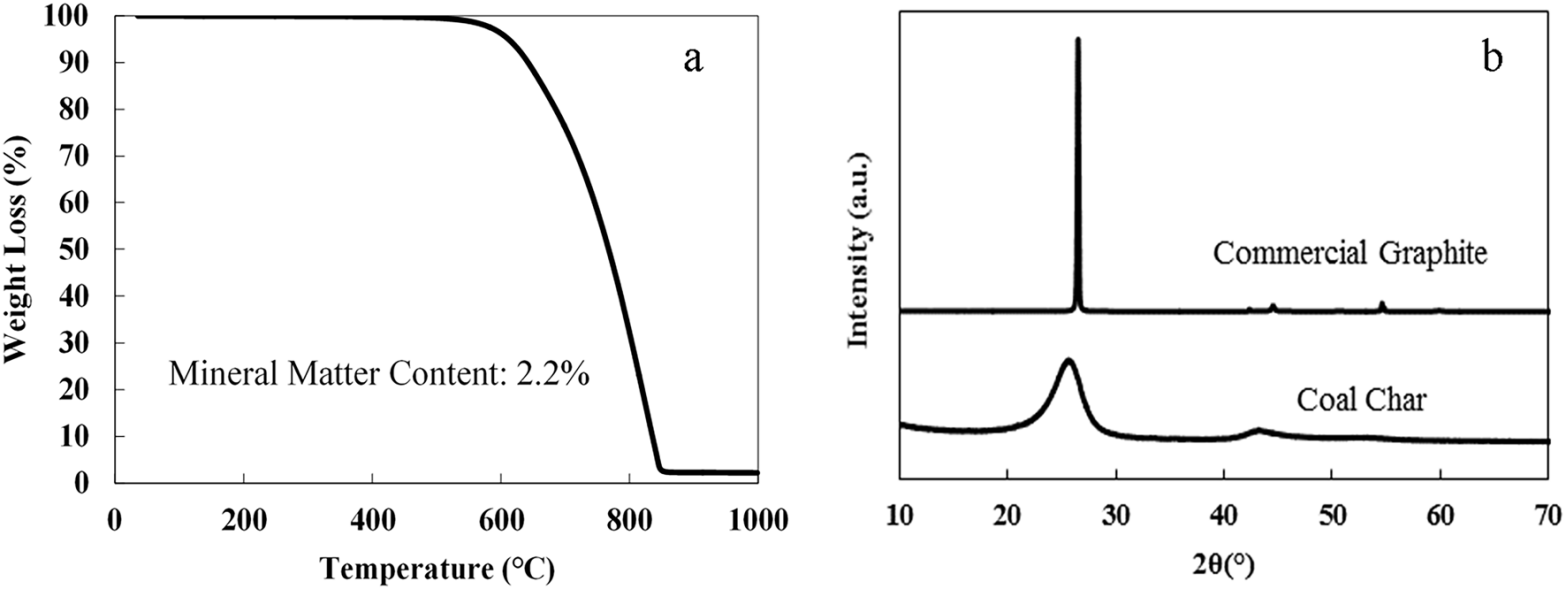
(**a**) TGA in air of Blue Gem coal char precursor; (**b**) XRD profiles of Blue Gem coal char precursor (bottom) and commercial graphite for comparison (top).

The composition of coal char was analyzed by SEM/EDS, which revealed carbon is the primary element (93.6 at.%) and oxygen is the second most abundant element (5.0 at.%), whereas other elements such as Al, S, Si, and Fe are also found at concentrations less than 1.0 at.% (Table 2). The morphology of the coal char characterized by SEM in Fig. 1 displays the layered structure formed from stacking micron-sized crumpled carbon sheets. The ash content of coal char was analyzed with TGA in air, which gives 2.2% as seen in Fig. 2a. The XRD pattern of coal char in Fig. 2b exhibits a broad, but intense, 002 peak centered at 25.8°, suggesting the high degree of order of stacked carbon layers, similar to the layered structure in graphite, but at a larger *d* spacing. The highly ordered, layered structure of Blue Gem coal char makes it an appropriate feedstock candidate to produce carbon nanomaterials by a mechanical exfoliation processes.

**Table 2:** Composition of blue gem coal char measured by EDS analysis.

Element	Percentage (atomic%)
C	93.6%
O	5.0%
Al	0.9%
S	0.2%
Si	0.2%
Fe	0.1%

### Synthesis of carbon nanomaterials from coal char

**Figure 3:**
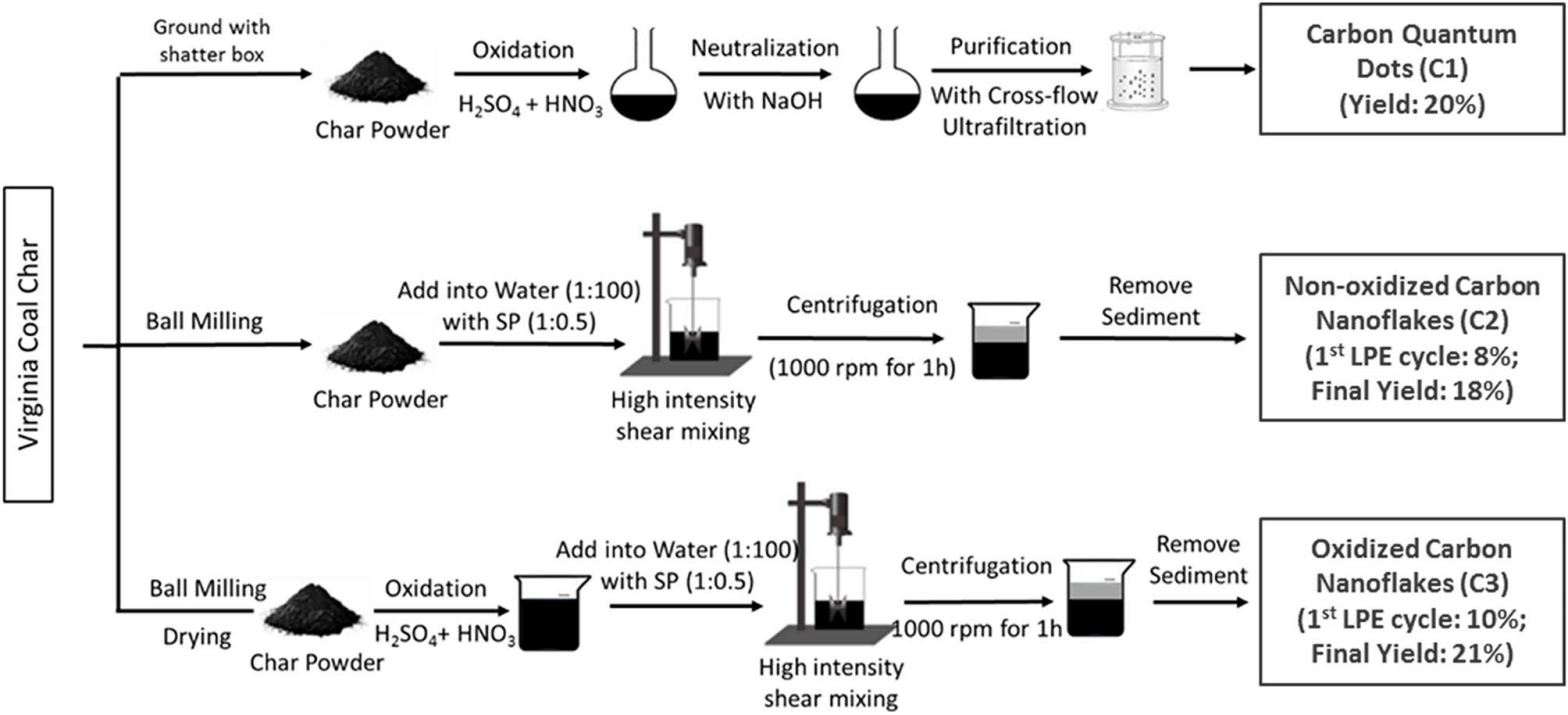
Schematic illustration of synthesis procedure of C1, C2, and C3.

The synthesis procedures of C1, C2, and C3 are schematically illustrated in Fig. 3, in which C1 was synthesized by chemical oxidation using a mixture of concentrated sulfuric and nitric acids, whereas C2 and C3 were synthesized by LPE^28^. The coals and coal char are well known to contain nanometer-sized crystalline graphitic carbon domains with defects that are linked by aliphatic amorphous carbons^29^.

During the production of C1 samples, heating the coal char in concentrated sulfuric and nitric acids initiates exfoliation of the nanometer-sized crystalline graphitic domains into small single sheets of graphenic carbon. The acid treatment also oxidizes most of the aliphatic carbon attached to these domains into CO_2_ and partially oxidizes the edges of the exfoliated graphenic sheets to form hydroxyl and/or carboxyl functional groups. The total diameter of the sheets is usually 2–4 nm. The C1 samples are referred to in the literature and this paper, as carbon quantum dots, also called graphene quantum dots^29^. After acid oxidation, neutralization, and purification steps were carried out to remove excess acids and byproducts. The obtained C1 has a production yield of approximately 20 wt.%, which is comparable with previous reports^29^.

**Figure 4:**
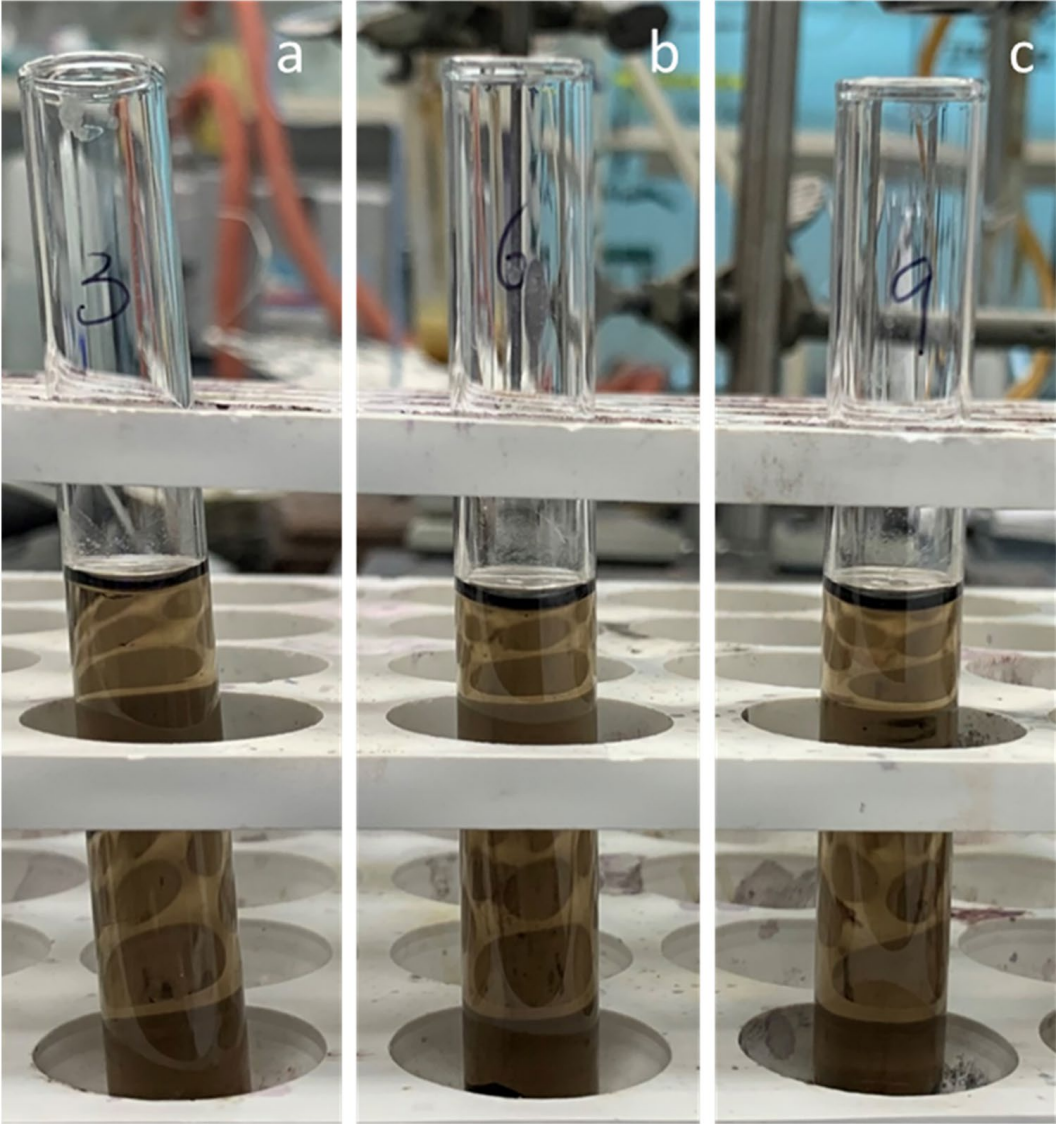
Photographs of C2 dispersed in cement pore solution (**a**) 30, (**b**) 60, and (**c**) 90 days after C2 was first added to the solution. The dispersion of C2 remained stable for at least 90 days and no visible change or precipitation of the carbon additives was observed. Cement pore solution was extracted from cement paste through centrifugation.

For the synthesis of C2 and C3, we take advantage of the layered structure of the coal char feedstock and use an LPE method to exfoliate the coal char into carbon nanoflakes^28^. The wet ball-milling step reduces the size of the coal char particles down to a few microns (1.0–5.0 μm) to facilitate the exfoliation of coal char in the subsequent high-shear exfoliation step. For C2, the ball-milled coal char was mixed with SP solution before liquid-phase high-shear exfoliation, which allows SP to adsorb on the surface of exfoliated carbon nanoflakes, stabilizing them in water, as well as in the cement pore solution later, and prevents them from restacking after exfoliation. The LPE of coal char was achieved by high-shear mixing at a speed (5000 rpm) at which coal char particles were trapped in the narrow space between the rotor blade and stator of the mixer head, and the shear forces developed in the liquid separated the carbon layers of coal char particles^18^. After that, centrifugation at low 1000 rpm was applied to separate the freshly exfoliated carbon nanoflakes and unexfoliated coal char particles. Subsequently, the supernatant containing fresh-exfoliated carbon nanoflakes was collected. The obtained C2 solution was highly stable, not only in water, but also in the cement pore solution, for at least 90 days, requiring only a mild bath-sonication for 10 min before use. This ensures the good dispersibility of C2 in the cement matrix during the curing process (Fig. 4). The exfoliation yield of C2 was 8%, which is significantly higher than the LPE of graphite which is only ~ 0.2%^18,30^. Moreover, unexfoliated coal char collected from the centrifugation step was reused as the feedstock for another cycle of LPE, with a slightly reduced exfoliation yield of 6% for the second cycle. The total exfoliation yield after 3 LPE cycles reached up to 18%.

Similar to C2, C3 was also prepared by LPE. However, an additional step of mild chemical oxidation of ball-milled coal char with a mixture of sulfuric and nitric acid at room temperature was carried out prior to high-intensity shear mixing. This was to introduce oxygen functional groups on the surface and edge of carbon nanoflakes and improve dispersibility in water. The exfoliation yields of C3 were 10%, 7%, and 4% for the first, second, and third LPE run, respectively. The total exfoliation yield after three LPE cycles reached up to 21%, which was slightly higher than observed for C2 samples.

### Characterization of carbon nanomaterials

**Figure 5:**
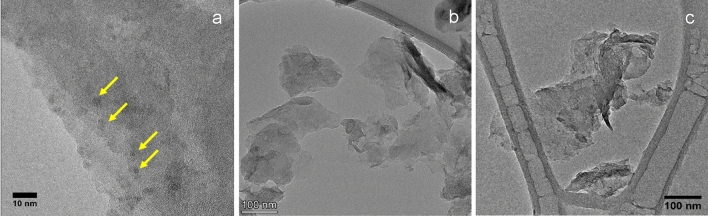
TEM images of (**a**) C1, (**b**) C2, and (**c**) C3. The yellow arrows in (**a**) highlight the carbon quantum dots (C1). Scale bar: (**a**) 10nm, (**b**) 100nm, (**c**) 100nm.

The three carbon nanomaterials were characterized by SEM, TEM, and XPS. Based on the TEM results (Fig. 5a), C1 consisted of small carbon quantum dots that are 2–4 nm in lateral size and only a single carbon layer in thickness. It was heavily covered with oxygen groups, as indicated by the low C/O ratio measured by XPS (Table 3).

**Table 3:** Comparison of coal-based carbon nanomaterials*

	C1	C2	C3
Lateral size (nm)	2–4	70–500	70–500
Carbon layers	1	3–5	3–7
Aspect ratio	2–4	50–100	50–100
C/O ratio	1.92	30.2	18.8

*The lateral size and carbon layers in Table 3 are based on statistical TEM analysis (see SI for TEM images and details). The C/O ratio is based on XPS analysis.

**Figure 6:**
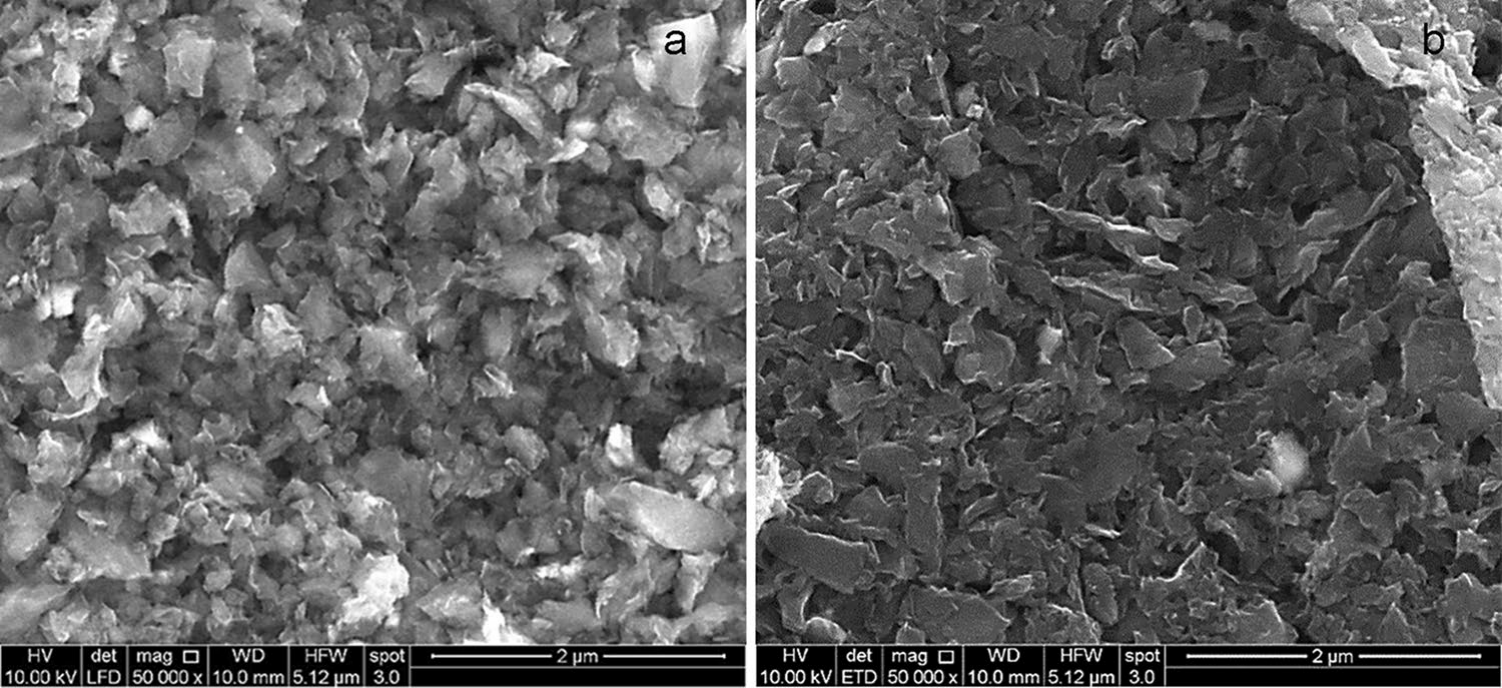
SEM images of (**a**) C2 and (**b**) C3. Scale bar: (**a**) 2  µm, (**b**) 2  µm.

In contrast, C2 and C3 produced using the LPE method are 2D nanoplatelets (Fig. 5b,c). Based on the SEM images (Fig. 6) and quantitative TEM image analysis of C2 and C3 (Table S1), their lateral sizes and thicknesses were in the range of hundreds of nm and < 10 layers, respectively (Table 3). The aspect ratio is calculated by dividing the lateral size by the thickness, assuming each carbon layer has ~ 1 nm thickness. The C/O ratio in Table 3 was calculated based on XPS analysis. C2 and C3 exhibited the 2D nanoplatelet morphology, with graphitic and amorphous layers observed under high-resolution TEM (Fig. S1, S2). They both have a median lateral size of 70–500 nm (Table S1) and 3–7 stacked carbon layers. The largest difference between C2 and C3 samples was that the surface of the C3 was moderately oxidized, resulting in a lower C/O ratio than C2 (Table 3). The purpose of the addition of oxygen groups on C3 was to examine how functional groups affect their reinforcing effects within the cement matrix. The lateral size and thickness of C2 and C3 are comparable with some of the graphene nanoflakes produced using LPE from graphite^30^, which illustrates that using coal char can produce carbon nanoflakes at a much higher yield and with similar morphology to those synthesized from graphite.

### The application of coal-based carbon nanomaterials in cement paste

#### Mechanical properties

The general physical morphology of the three types of carbon nanomaterials synthesized from coal was investigated. C2 and C3 resemble the properties of graphene nanoflakes reported in the literature for the enhancement of cementitious composites, whereas the much smaller nm-sized C1 has not been reported in the literature for this application, to the best of our knowledge.

**Figure 7:**
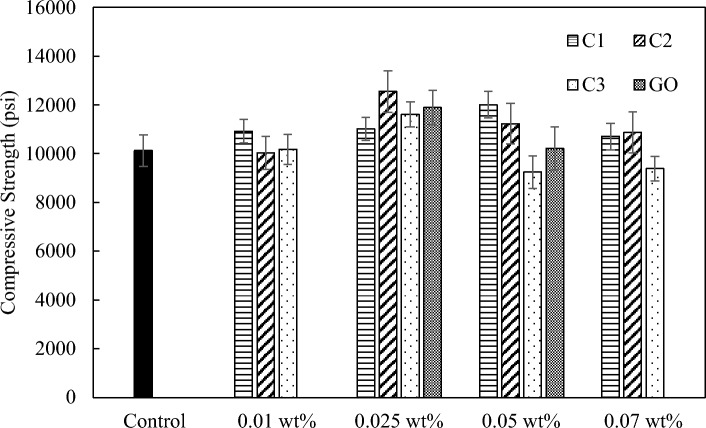
Compressive strength with C1, C2, and C3 at loadings between 0.01 and 0.07 wt.% in comparison with the control neat cement sample and commercial GO additive.

All three coal-based carbon nanomaterials demonstrate the ability to improve the compressive strength of the cement paste (Fig. 7). The optimum loading for the carbon quantum dots (C1) was 0.05 wt.%, enabling an improvement of 19% in compressive strength compared to the neat cement used as a control sample. The non-oxidized carbon nanoflakes (C2) performed best at a loading of 0.025 wt.% which imparts a 24% improvement over the control sample. The oxidized carbon nanoflakes (C3) have an optimum dosage at 0.025 wt.%, leading to an improvement of 15% in compressive strength. The highest improvement seen for the compressive strength of the commercial GO samples was 17% which occurs at the loading of 0.025 wt.%. Higher loadings (C1 at 0.07 wt.%, C2 at 0.07 wt.%, and C3 at 0.05 wt.% and 0.07 wt.%) tend to result in a decreased enhancement effect, which is likely due to the challenge associated with maintaining good dispersion of nanomaterial additives in cement matrix and the reduced workability of the cement^31,32^. Previous studies reported gradually decreased flowability of cement composites with increasing dosage of graphene additions. For example, Shuang et al. demonstrated that the viscosity of the cement paste increases by 19%, 28%, and 41% at 0.03 wt.%, 0.06 wt.%, and 0.09 wt.% of GNP, respectively^31^. Another study also noted that the flowability of cement paste decreases by 17.4% and 39% by adding 0.2 wt.% and 0.4 wt.% of GNP in cement^32^. Among the three carbon nanomaterials, C2 shows the largest reinforcing effect on compressive strength, and all three coal-derived carbon nanomaterials have better or comparable improvements compared to the commercial GO, as shown in Fig. 7.

**Figure 8:**
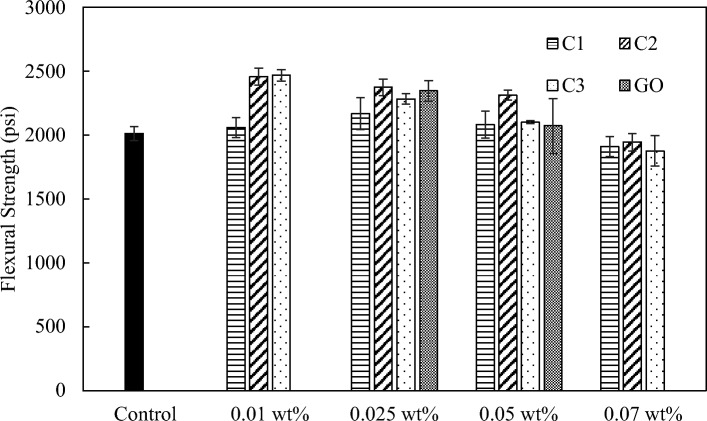
Flexural strength with C1, C2, and C3 at loadings between 0.01 and 0.07 wt.% in comparison with the control neat cement sample and commercial GO additive.

All three carbon additives were added to cement at different dosages and measured for their flexural strength under a three-point bending test (Fig. 8). The largest improvement of flexural strength of approximately 23% was observed with C2 and C3, both at 0.01 wt.%, compared to the control sample. The improvement in flexural strength with C2 and C3 decreased with increasing dosages. In contrast, the optimum dosage for C1 was at 0.025 wt.%, leading to an improvement of flexural strength by approximately 8%, which was more modest compared to C2 or C3. Again, all three coal-derived carbon additives demonstrated better or similar enhancement effects compared with commercial GO in our experiments.

The literature reported that adding graphite-derived graphene materials (including GNP or GO) imparts improvement to the compressive and flexural strength of cement composites (Table S3). The typical improvement of compressive and flexural strength reported was in the range of 10–40% and 10–60%, respectively. These reported values are comparable to the enhancement caused by our coal-based carbon additives. Given the fact that coal feedstocks are much more abundant and economical, and that the production yield is much higher, coal-derived carbon nanomaterials have obvious advantages over graphite based carbon additives for cement composite applications.

#### Durability

In addition to mechanical properties, improving the durability of cementitious materials can also have a significant impact by ensuring structural integrity and reducing environmental footprint. The durability of concrete governs the service life of concrete structures and thus has a significant impact on the life-cycle cost. The durability can be increased by reducing the ingression of both liquids and gases into concrete, and it highly depends on the permeability and porosity of the cement matrix^33^. Studies have found that the graphene family of materials can refine the pore structure, increase the durability of cement-based composites, and extend the lifetime of the concrete structure^16,17,34–37^.

**Figure 9:**
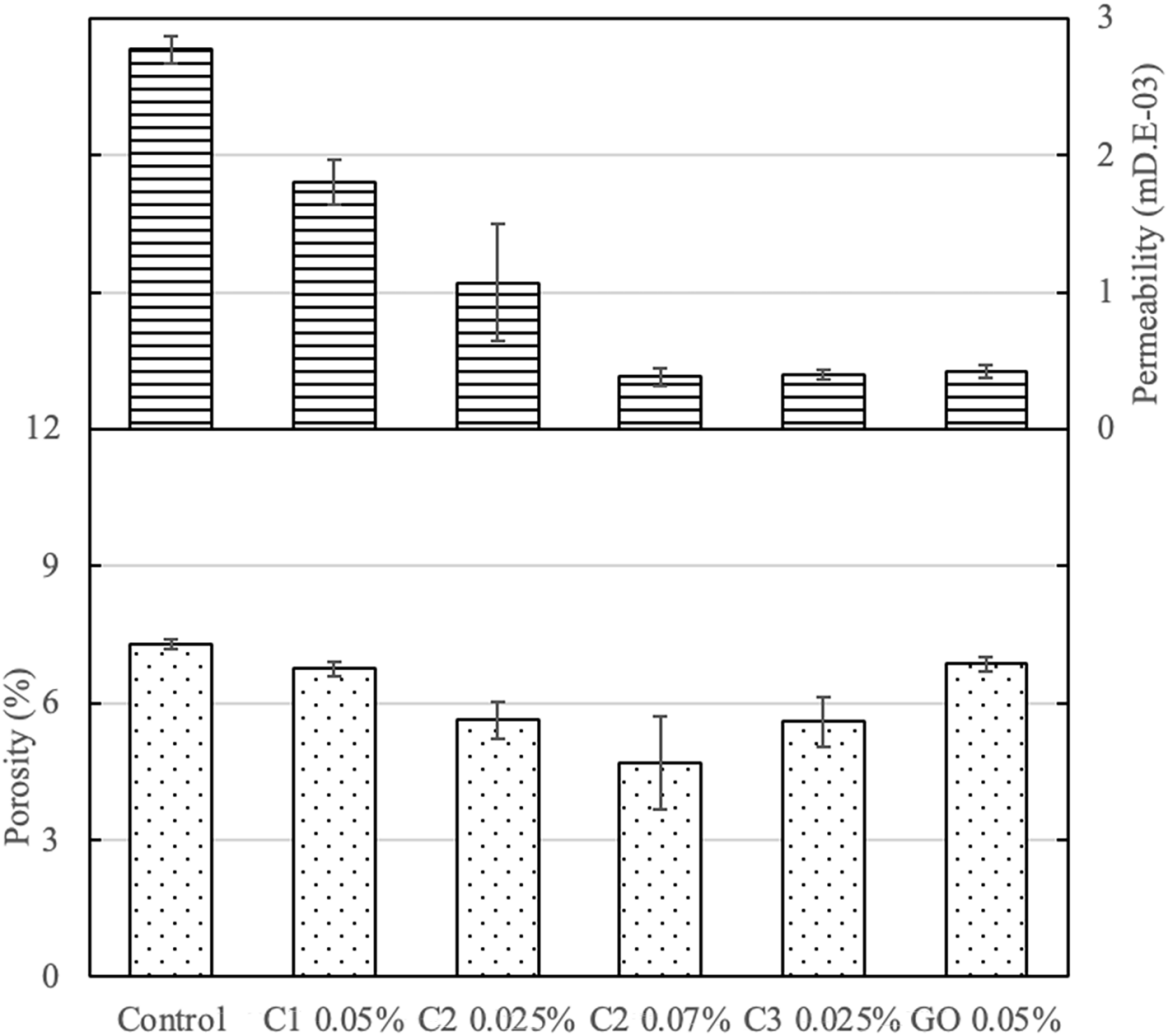
Permeability and porosity of cement pastes with different coal-based carbon nanomaterials, as well as control neat cement and commercial GO-added samples.

Figure 9 shows the effect of coal-based additives on the permeability and porosity of cement. Each of the C1, C2, and C3, as well as the commercial GO, additives reduced the permeability of the cementitious composite. C2, at 0.07 wt.%, and C3, at 0.025 wt.%, demonstrated the largest reduction in permeability by approximately 86%. The porosity of the cement composites was not as dramatically impacted, but C1 at 0.05 wt.%, C2 at 0.025 wt.%, and C2 at 0.07 wt.%, still led to a pronounced reduction in porosity by 8%, 23%, and 36%, respectively. The addition of nanomaterials likely had a significant impact on the nano-sized pores, but a limited impact on the micro-scaled pores. Therefore, the total porosity was expected to be only partially reduced as a result of the reduction of nano-pores, which agrees with the observed test results (Fig. 9).

**Figure 10:**
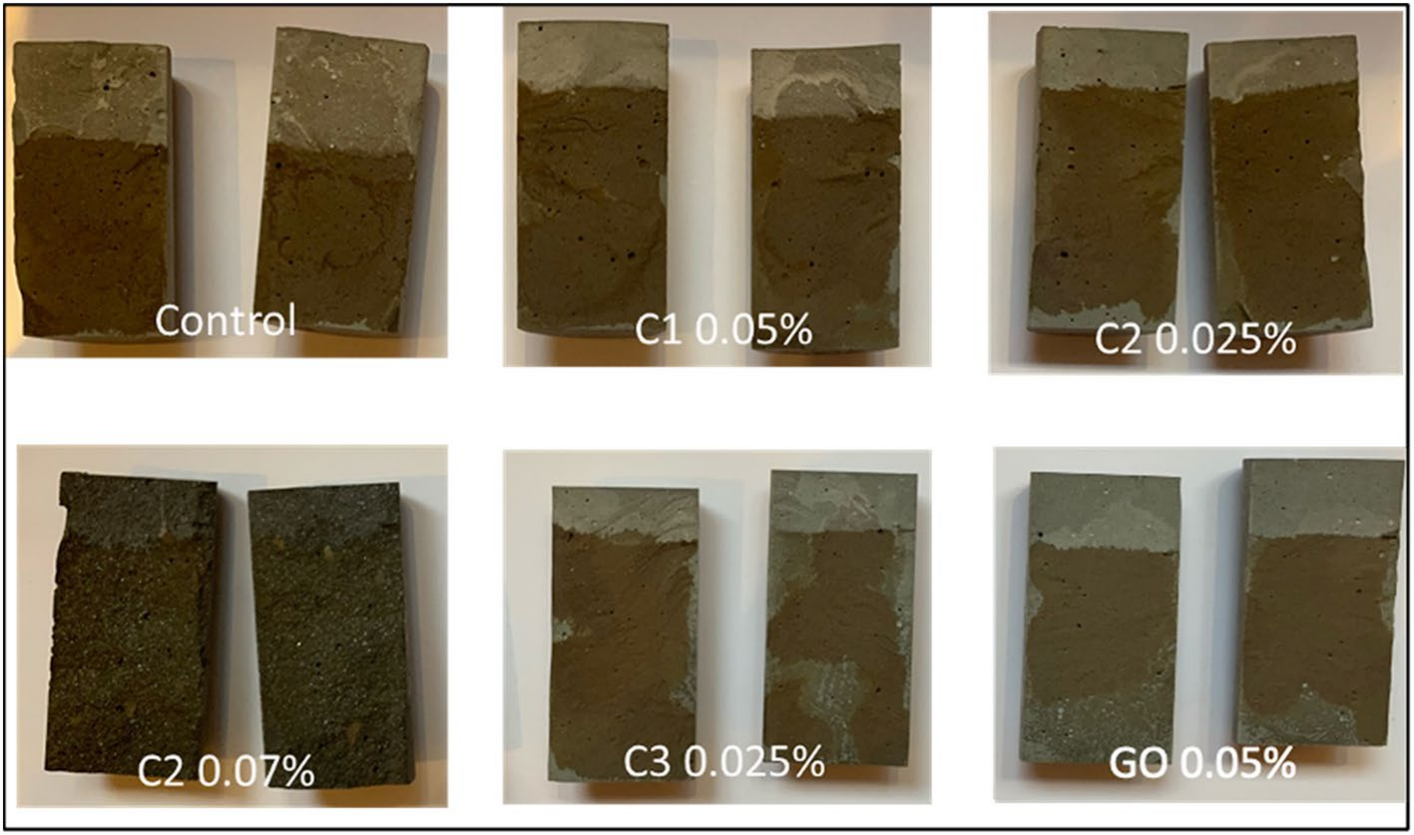
Photographs of chloride penetration test samples with neat control cement, and cement composite with carbon additives at different dosages.

**Figure 11:**
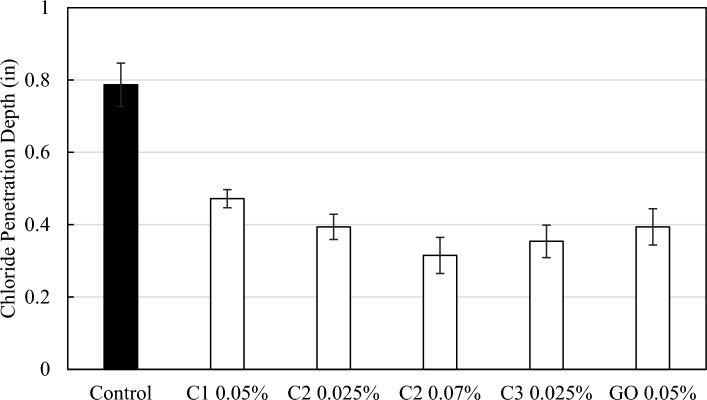
Chloride penetration depth of cement pastes with three different coal-based carbon nanomaterial additives as well as control neat cement and commercial GO-added samples.

The effectiveness of carbon additives for improving the anti-corrosion and durability properties of cementitious composites were further evaluated with chloride ion penetration measurements. The measurement of chloride penetration depth is a good indication of the resistance to chemical ingression. All three nanomaterial-enhanced samples have reduced chloride penetration depths in comparison to the neat cement sample (Figs. 10, 11). C2 at 0.07 wt.% enabled the largest reduction in chloride ion penetration by 60%. C1 at 0.05 wt.% results in 40% reduction and C3 at 0.025 wt.% exhibited 55% reduction in chloride penetration depths. As a further comparison with commercially available carbon additives, commercial GO-added cement paste was also tested for chloride ion penetration and it led to 50% reduction.

The effect of graphite-based graphene materials, including GNP as well as GO, on the porosity and durability of cementitious composites has been studied. Baomin et al. reported a reduction of 40% in total porosity with 0.06 wt.% of graphite-based GNPs^35^. Another study found the total porosity can be reduced by 13.8% at the age of 28 days with the addition of 0.04 wt.% of GO^36^. While for permeability, a reduction in the relative permeability coefficient of 80.2% was observed with 0.06 wt.% of graphite-based GO^37^. Furthermore, a study observed that the GO at 0.1 wt.% led to a decrease of the chloride penetration of cement paste by 75%^16^. Du. et al. reported an 80% reduction in chloride penetration depth with 0.5 wt.% of GNPs^17^.

It is evident that the coal-based carbon nanomaterials studied here demonstrate comparable, or even superior, capabilities for reducing cement’s porosity and permeability compared to those reported in the literature using graphite-derived additives. This work confirms that improved effectiveness in delaying the ingression of water or corrosive chemicals can be achieved using carbon additives synthesized from inexpensive coal feedstocks.

In Section "The application of coal-based carbon nanomaterials in cement paste", it was shown that coal-derived carbon additives can enhance the mechanical properties and improve the durability of cement paste by increasing the compressive strength and flexural strength and reducing the permeability, porosity, and chloride penetration depth. At a low loading of 0.025 wt.% of coal-derived carbon additives, the compressive and flexural strength can be increased by > 20%. In contrast, the permeability can be reduced by up to one order of magnitude at a much higher loading of 0.07 wt.%. The reason for this observation is that the mechanical properties are more sensitive to the dispersion of the additives within the cement matrix, as well as the workability of the composite materials. High loadings of nano-additives lead to more difficulty in uniform dispersion, and tend to cause deteriorated workability of the composite material due to the large amount of the dispersing agent (superplasticizer in our experiment) required at higher loadings^31,32^. On the other hand, permeability/porosity and the chloride penetration are less sensitive to the dispersion condition of the nanomaterials, and demonstrate better performance with higher loadings.

### Coal-derived carbon nanomaterials as additives for concrete

**Figure 12:**
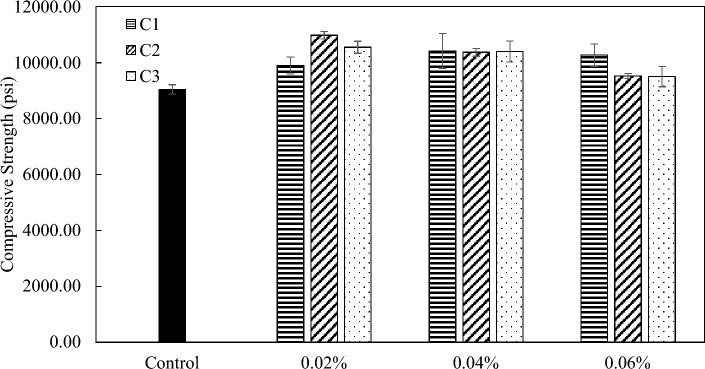
Compressive strength of mix 1 with C1, C2, and C3 at loadings at 0.02 wt.%, 0.04 wt.%, and 0.06 wt.%.

**Figure 13:**
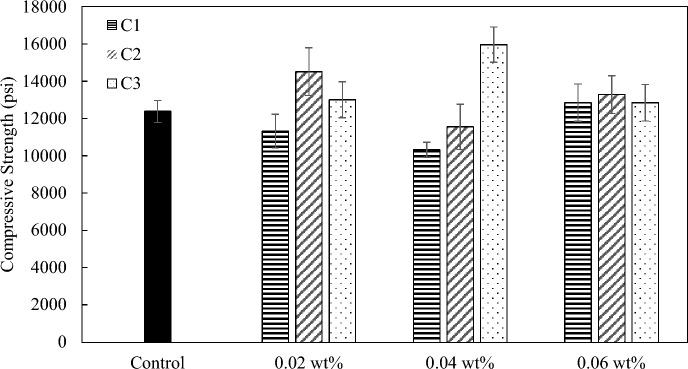
Compressive strength of mix 2 with C1, C2, and C3 at loadings at 0.02 wt.%, 0.04 wt.%, and 0.06 wt.%.

The effect of coal-based carbon additives on the mechanical properties of concrete was also studied. Two mix designs that correspond to normal strength and high-strength concrete were prepared. For mix 1, all three carbon nanoadditives were able to improve the compressive strength at varying degrees (Fig. 12). The largest improvement of C1, C2, and C3 were 15%, 21%, and 17%, respectively. The optimum additive dosage for C2 and C3 occur at 0.02 wt.%, and the optimum dosage for C1 at 0.04 wt.%. For mix 2, C1 showed a 4% increase in compressive strength at the optimum loading of 0.06 wt.%. C2 showed a 17% increase in compressive strength at 0.02 wt.%. C3 had the largest reinforcing effect of 29% increase in compressive strength at a loading of 0.04 wt.% compared to the control sample used for mix 2 concrete materials s (Fig. 13). The oxidized carbon nanoplatelet additives (C3) might have a better dispersion in mix 2 concrete compared to mix 1 concrete due to the introduction of silica fume, which was expected to consume some amount of the calcium hydroxide in the cement pores and reduce the cross-linking effect. At higher dosages, all samples have little effect on compressive strength, which was consistent with the observation in cement paste samples (Fig. 7). The reinforcing effect of carbon nanoadditives (GNP or GO) as well as other nanomaterials (nanoclay) on concrete was also studied by other researchers, with 25–45% of improvement in mechanical properties reported (Table S3).

**Figure 14:**
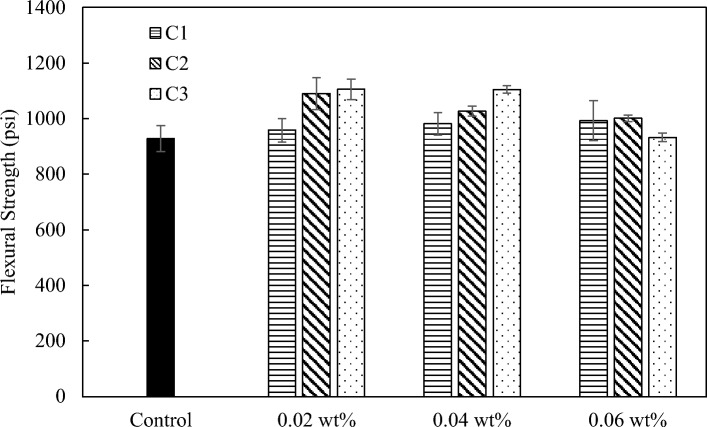
Flexural strength of mix 1 with C1, C2, and C3 at loadings at 0.02 wt.%, 0.04 wt.%, and 0.06 wt.%.

**Figure 15:**
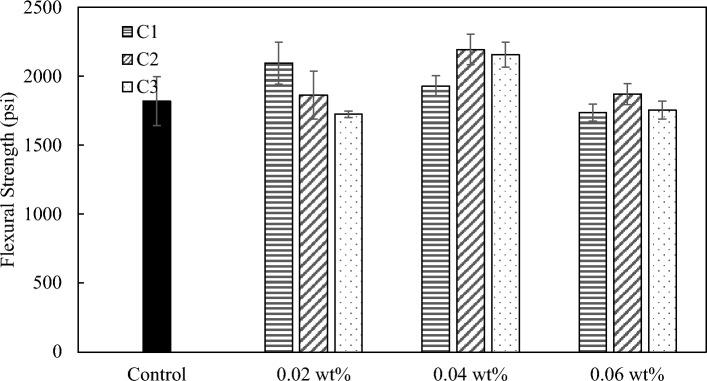
Flexural strength of mix 2 with C1, C2, and C3 at loadings of 0.02 wt.%, 0.04 wt.%, and 0.06 wt.%.

For flexural strength, all three coal-based carbon nanomaterials can effectively reinforce the concrete. For mix 1, the largest improvement of C1, C2, and C3 were 7%, 17%, and 19%, respectively (Fig. 14). For mix 2, the largest improvement of C1, C2, and C3 were 15%, 21%, and 18%, respectively (Fig. 15). C1 has less reinforcing effect on flexural strength compared to C2 and C3 for both mixes, while both C2 and C3 demonstrate promising reinforcement effect on both compressive and flexural strength of concrete.

### Characterization of hydration products

Fundamentally, the mechanical properties of cement composites are directly related to the microstructure and composition of cement hydrates, in particular, calcium silicate hydrate, C–S–H, and Portlandite, Ca(OH)_2_. The amorphous C–S–H is considered as the strongest phase in hardened cement paste, and Ca(OH)_2_ is considerably weaker. In this research, the proposed reinforcement mechanisms are: (1) the nanoadditives refine the nanostructure of the hydration products, which leads to enhancement of the mechanical properties; (2) the large specific surface area of the carbon nanomaterials (especially C2 and C3) make them act as the nucleation site, which could facilitate the hydration reactions and lead to improved mechanical properties; and (3) the microstructure is refined with the addition of nanomaterials, where the nanoadditives fill the pores leading to a denser microstructure with lower total porosity. The total porosity has been examined in the permeability/porosity test in the previous section and proves the densification effect with the addition of carbon nanomaterials. In order to further understand the mechanism of property reinforcement of cement composites, characterization of hydration products was carried out using XRD and DSC-TGA.

**Figure 16:**
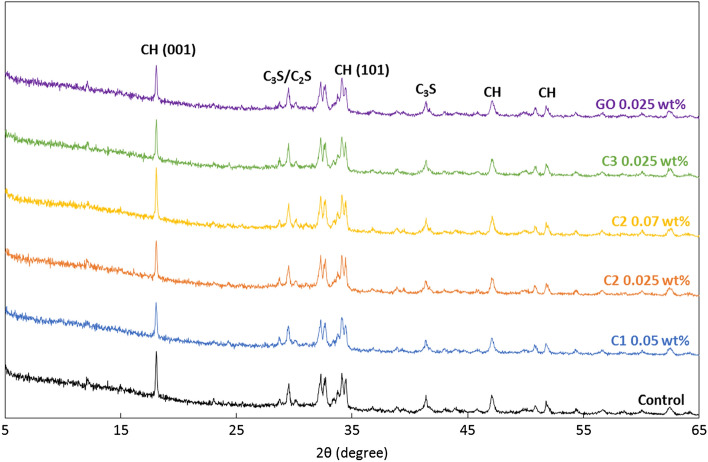
XRD pattern of cement pastes with three different coal-based carbon nanomaterial additives as well as control neat cement and commercial GO added samples.

The XRD patterns of the cement samples at the age of 28 days are presented in Fig. 16. Typical cement hydration products such as ettringite, Portlandite, tricalcium silicate (C_3_S), dicalcium silicate (C_2_S) were detected for all samples, and the addition of carbon nanoadditives neither added nor eliminated any specific hydration products. The amorphous C–S–H was not detectable with XRD. On the other hand, the crystal orientation of Portlandite (i.e., crystalline calcium hydroxide) changed with the incorporation of carbon additives, which was directly correlated with the strength of cement-based materials: more random crystal orientation led to higher strength. The crystal orientation can be estimated by the orientation index value of CH crystals, *R* with the following equation:

1 $$R=1.35\times {I}_{001}/{I}_{101}$$

where *R* is defined as 1.35 times the ratio of the peak intensities at 001 (at 18.0°) and 101 (at 34.1°) of XRD pattern for CuKα radiation. This relation is derived and explained in detail in^38^. The *R* values for different cement samples are listed in Table 4. When the CH crystals are randomly oriented, *R* = 1; when the arrangement of CH crystals are aligned, *R* > 1; and the larger *R* is, the more aligned the crystal orientation is.

**Table 4:** Crystal index *R* of portlandite of cement paste samples with different coal-derived carbon additives, commercial GO additive and the neat control sample.

	Control	C1 0.05 wt.%	C2 0.025 wt.%	C2 0.07 wt.%	C3 0.025 wt.%	GO 0.025 wt.%
Orientation Index of CH Crystal, *R*	2.30	1.84	1.81	2.18	1.89	1.74

In this work, the *R* values were reduced for C1, C2, and C3, indicating that the carbon nanomaterial additives led to a more random orientation of the CH crystals, thus preventing the growth of the CH crystals. Not surprisingly, the reduction in *R* (Table 4) followed a similar trend to the compression test results (Fig. 7): C2 at 0.025 wt.% with the largest increase in compressive strength had the largest decrease of *R*, C1 at 0.05 wt.% and C3 at 0.025 wt.% had smaller yet still obvious decrease of *R* corresponding to smaller and measurable increase in compressive strength, and C2 at 0.07 wt.% had the least decrease of *R* and also the smallest enhancement in compressive strength compared to the neat control sample without any carbon additives. The commercial GO also had a reduced *R* value compared to the control sample which was consistent with the observations reported in^39–41^. In^39^, the authors observed the lowered CH orientation index after adding GO at both 1 day (from 1.75 to 1.56) and 7 days (from 4.49 to 4.39) of cement age, which resulted in the refined microstructure and increased compressive strength of their cement samples (12% improvement at 7 days compared to plain cement). In^40^, the authors found that 0.025 wt.% of graphene sheets were able to reduce the CH orientation index from 1.63 to 1.43 at 28 days, with which the compressive strength as increased by 10% and the flexural strength was increased by 16% compared to plain cement. In^41^, the authors found that 1 vol.% or 2 vol.% of graphene addition reduced the CH orientation index from 3.05 to 1.88 at 90 days. The authors also observed enhancements on compressive strength by 154% and on flexural strength by 21% with the addition of 2 vol.% of graphene at 90 days of cement age.

**Figure 17:**
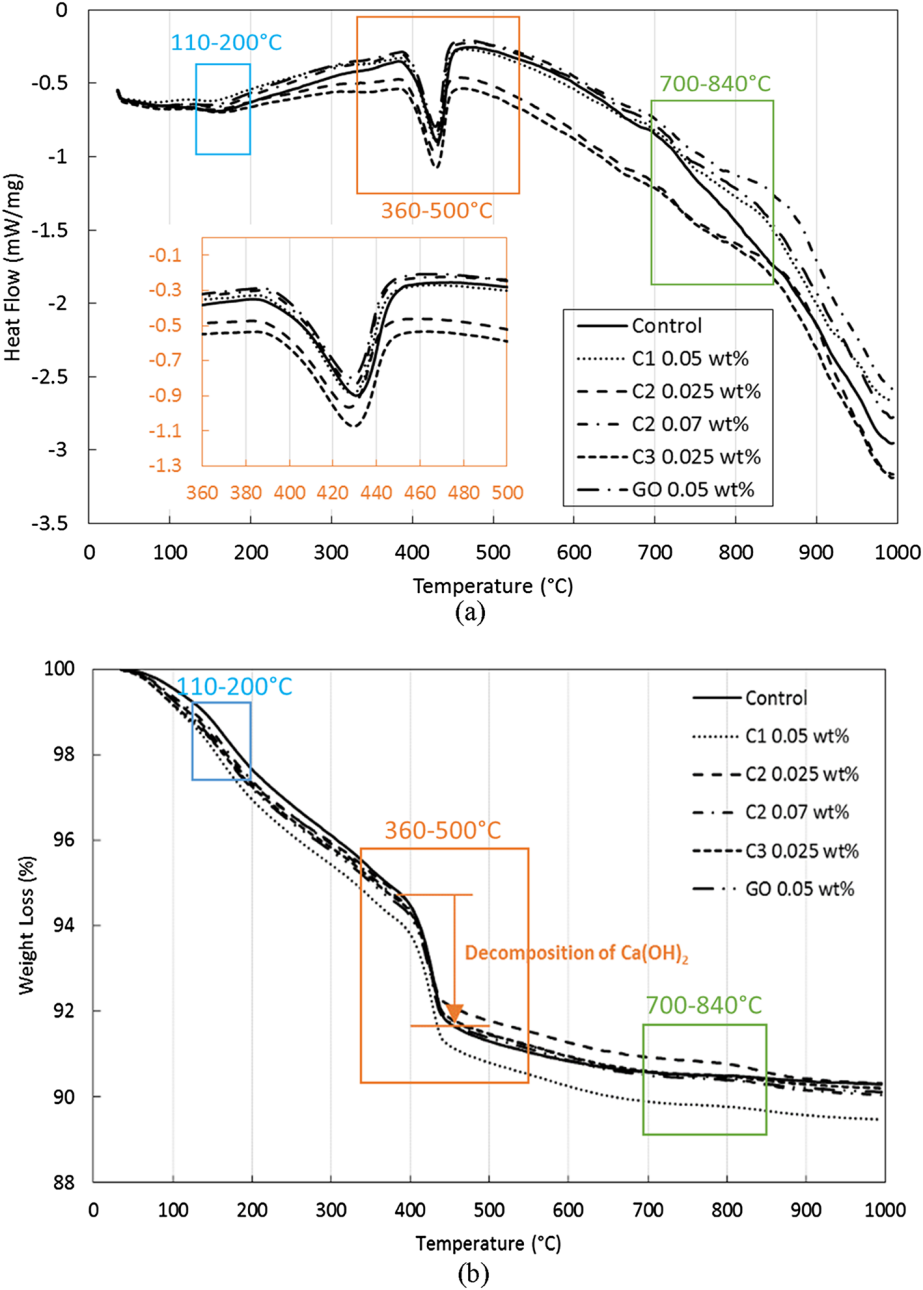
(**a**) DSC and (**b**) TGA curves of cement pastes with three different coal-based carbon nanomaterial additives as well as control neat cement and commercial GO added samples. The blue, orange, and green boxes in (**a**) highlights the three temperature ranges where significant reactions occur. The inset in (**a**) shows more details during the decomposition of Portlandite.

A simultaneous DSC-TGA measurement was conducted on cement samples at the age of 28 days. DSC measures the difference in heat (energy) needed to keep both the reference material and the sample at the same temperature. It is used to study the occurrence of glass transition, crystallization, oxidation, and chemical reactions. TGA measures the change of mass in terms of temperature while the sample is subjected to a controlled temperature. The heat flow (in mW/mg) and weight loss (in %) obtained for the cement paste samples at a given temperature are shown in Fig. 17. In Fig. 17a, three significant endothermic heat flow peaks can be observed. The first peak in the temperature range from 110 to 200 °C corresponds to the dehydration reactions caused by the loss of water mainly from calcium silicate hydrates^42^. The reactions in this temperature range are associated with a subtle weight loss of ~ 3% (see Fig. 17b for detail). The second peak in the temperature range from 390 to 450 °C represents the decomposition of Portlandite (see Fig. 17a) and it is accompanied by a significant weight loss of 7% (see Fig. 17b). The peaks in the temperature range 700–840 °C correspond to the decomposition of calcite (CaCO_3_), which is associated with a very minimal weight loss as shown in Fig. 17b. The Ca(OH)_2_, CaCO_3_, and C–S–H contents were calculated by the following equations^43^:

2 $${\text{LOI}}\left( {{\text{CH}}} \right)\left( \% \right) \, = {\text{ Ca}}\left( {{\text{OH}}} \right)_{{2}} {\text{mass loss from 39}}0 - {45}0^\circ {\text{C}}/{\text{sample mass at 1}}000^\circ {\text{C}} \times {1}00$$

3 $${\text{LOI}}\left( {{\text{CC}}} \right)\left( \% \right) \, = {\text{ CaCO}}_{{3}} {\text{mass loss from 7}}00 - {84}0^\circ {\text{C}}/{\text{sample mass at 1}}000^\circ {\text{C}} \times {1}00$$

4 $${\text{LOI}}\left( {{\text{CSH}}} \right)\left( \% \right) \, = {\text{ mass loss from 1}}0{5} - {1}000^\circ {\text{C}}/{\text{sample mass at 1}}000^\circ {\text{C}} \times {1}00 \, - {\text{ LOI}}\left( {{\text{CH}}} \right) \, - {\text{ LOI}}\left( {{\text{CC}}} \right)$$

where LOI(CH) is the percentage of Ca(OH)_2_ as measured by H_2_O loss in this temperature range in the TGA curve, LOI(CC) is the percentage of CaCO_3_ with loss of CO_2_ in the TGA curve and LOI(CSH) is the percentage of the C–S–H with H_2_O loss in the TGA curve.

**Figure 18:**
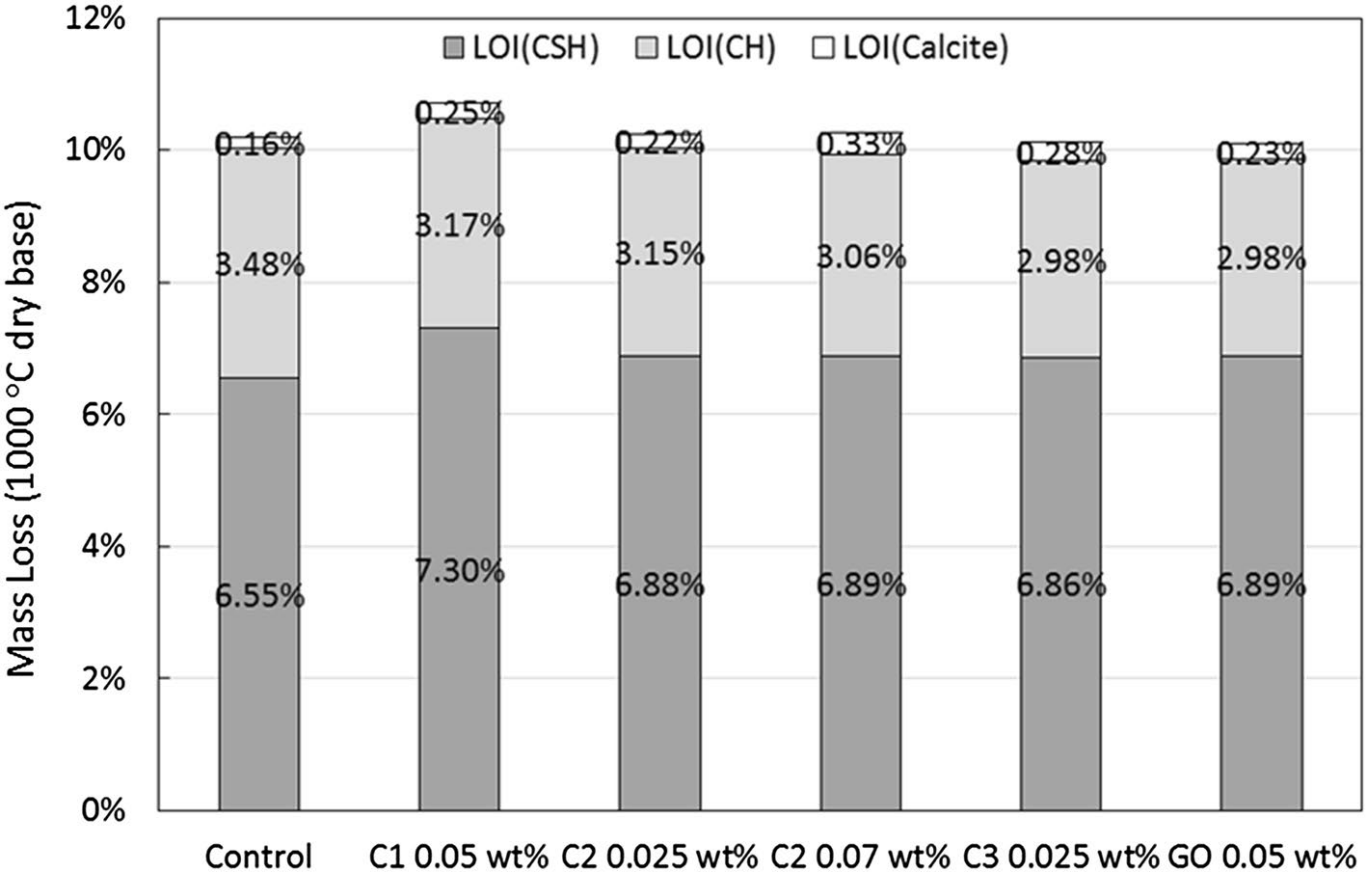
Mass loss of the hydration products for samples at 28 days of cure relative to the dry mass at 1000 °C.

The content of different hydration products, including C–S–H, Ca(OH)_2_, and CaCO_3_, for cement samples are presented in Fig. 18. The general trend for carbon nanomaterial added cement samples is that the amount of CH decreases, whereas the amount of both C–S–H and CaCO_3_ increases. The reduction of CH could be caused by the reaction of COO^-^ on the edge of carbon nanosheets with Ca ions to produce Ca(HCOO)_2_. The consumption of Ca(OH)_2_ can promote the hydration of cement grains and lead to increased amount of C–S–H and CaCO_3_. Among different carbon nanomaterials added cement samples, C1 at 0.05 wt.% has the largest increase in the amount of C–S–H by ~ 11% (from 6.55 to 7.30%), compared to the control sample. C2 at 0.025 wt.% and 0.07 wt.% and C3 at 0.025 wt.% result in a smaller increase of approximately 5% of the content of C–S–H. C1 is expected to have the most carboxyl groups (COO^-^) among the three different coal-based carbon nanoadditives because of its synthesis process (and as suggested by the lowest C/O ratio shown in Table 3), which is in agreement with the observations of the improvement on the C–S–H content. Because of the lack of the oxygen-rich functional groups, C2 and C3 lead to significantly reduced consumption of Ca(OH)_2_, and thus less promotion of C–S–H formation. The CH reduction effect has also been reported by other authors^44–46^. Wang^44^ calculated the amount of Ca(OH)_2_ from both DSC and TGA measurements, and identified the decreasing trend with increasing amount of GO added from 0.01 to 0.05 wt.%. The largest reduction on the amount of Ca(OH)_2_ occurs at 0.05 wt.% of GO addition, which is from 14.2 to 11.6% (Portlandite amount is converted from water loss associated with the dehydration of Portlandite). Mokhtar^45^ saw reductions on Ca(OH)_2_ within cement matrix at 28 days with the addition of graphene oxide nanoplatelets (GONPs) from 0.01 to 0.05 wt.%. The optimum reduction of approximately 4% compared to plain cement happens at 0.02 wt.% of GONPs. Krystek^46^ reported a reduction from 16.37 to 15.17% of the amount of Ca(OH)_2_ after adding GO at 0.05 wt.% and from 16.37 to 13.07% after adding electrochemically exfoliated graphene (EEG) at 0.05 wt.%. A similar conclusion was found in another study with the use of Raman and molecular dynamics simulation^47^.

Combining the results of XRD and DSC-TGA, we can conclude that the reinforcing mechanisms of the three coal-based carbon nanoadditives are slightly different. All three carbon nanomaterials can refine the crystalline structure of Portlandite, as indicated by the lowered CH crystalline index *R*, leading to increased compressive strength. In addition, the oxygen-rich C1 has the ability to promote the hydration process by consuming the calcium ions, which also leads to improved mechanical properties. On the other hand, based on the porosity/permeability test, C2 and C3 are better at refining the pore structure of the cement matrix, in which the additives can fill the cement pores and form a denser structure. With large aspect ratios, C2 and C3 are also much more capable at bridging micro-cracks, leading to improved flexural strength.

## Conclusion

In this paper, three coal-derived carbon nanomaterials, C1, C2, and C3 were synthesized and examined for their reinforcing effect in cement composites. The nanomaterials were characterized for size, morphology, and elemental composition. When incorporated into cement paste, the mechanical properties, including compressive strength and flexural strength were measured. The durability, including permeability/porosity and chloride penetration, was also evaluated. In addition, the three carbon nanomaterials were applied in concrete samples for their reinforcing effect on mechanical properties. At last, the reaction mechanisms of the three carbon nanoadditives were studied with XRD and DSC-TGA. The following conclusions were obtained:

1. All three types of coal-derived carbon nanomaterials enhance the cement’s compressive and flexural strength. The largest improvement for compressive strength was observed with C2 at 0.025 wt.%, representing a 24% increase compared to control cement sample. The largest improvement for flexural strength was observed with both C2 and C3 at 0.01 wt.%, and the enhancement was 23% compared to control samples.
2. All three types of coal-derived carbon nanomaterials reduced the gas permeability, gas porosity, and chloride ion penetration depth in cement samples. The largest reduction effect was found with C2 at 0.07 wt.%, which reduced the permeability, the porosity and chloride ion penetration by 86%, 36%, and 60%, respectively.
3. Both the compressive and flexural strength of concrete was improved with the addition of the three different carbon nanomaterials. For mix 1 concrete which in accordance with normal-strength concrete, the highest improvement of compressive strength (21%) was achieved with C2 at 0.02 wt.%, and the highest improvement of flexural strength (19%) was achieved by C3 at 0.02 wt.%. For mix 2 concrete which in accordance of high-strength concrete, the highest improvement of compressive strength (29%) was achieved with C3 at 0.04 wt.%, and the highest improvement of flexural strength (21%) was achieved with both C2 at 0.04 wt.%.
4. The improvement of mechanical properties and durability of cementitious composites using coal-derived carbon additives were comparable with, or better than, those reported in the literature with graphite-based carbon additives. The low cost and wide abundance of coal feedstocks and high production yield of additives from them could make large scale deployment of this technology more feasible. Additionally, graphite demand will be high in coming years to support battery markets^48^, which will further increase costs and reduce the supply of this more traditional feedstock, further supporting the benefits of utilizing coal.
5. During the study of the reinforcing mechanisms, all three types of coal-based carbon nanomaterials reduced the CH orientation index *R*, confirming their ability to refine the crystalline structure of the hydration products, which led to higher compressive strength. In addition, the oxygen rich C1 was found to promote the hydration reaction by consuming the Ca^2+^ and increasing the amount of C–S–H. On the other hand, C2 and C3, with high aspect ratio could fill the cement pores and form a denser structure. The larger C2 and C3 could also bridge microcracks leading to improved flexural strength. These mechanisms are consistent with what has been proposed for similar carbon-based nanoadditives.

### Supplementary Information

Supplementary Information.

## Data Availability

The datasets used during the current study available from the corresponding author on reasonable request.
