# Supplementary material for: High-performance cementitious composites containing nanostructured carbon additives made from charred coal fines
Source: Sci Rep. 2024 Apr 17;14:8912. doi: 10.1038/s41598-024-59046-y (PMC11024156; doi:10.1038/s41598-024-59046-y)
Supplement: Supplementary file 1 — Supplementary Information. [file 41598_2024_59046_MOESM1_ESM.docx]

**High Performance Cementitious Composite Using Coal-derived Carbon Additives**

Supplementary Information

1. Characterization of coal char

The mineral content

To get C1, the coal char was ground with the shatterbox for 3 minutes and then synthesized by chemical oxidation. The SEM characterization is conducted with the grounded coal char as shown in Fig. S1. To get C2 and C3, the coal char was ground with the shatterbox for 3 minutes, and then ball-milled for 2 hours, followed by the liquid-phase exfoliation. SEM characterization is conducted with the ball-milled coal char as shown in Fig. S2.

| 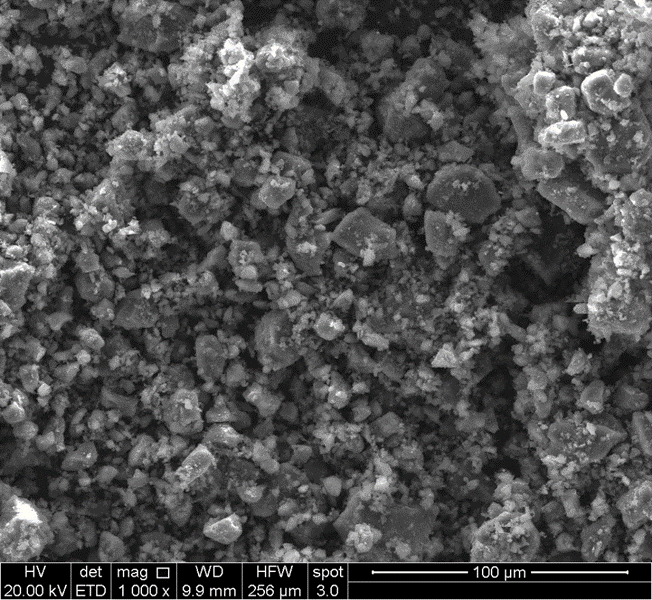 | 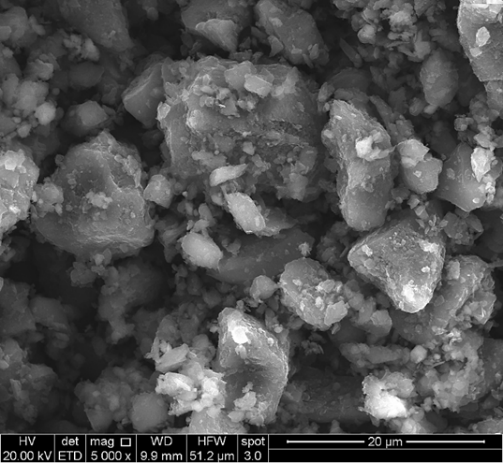 |
| --- | --- |

Figure S1. SEM images of grounded coal char

| 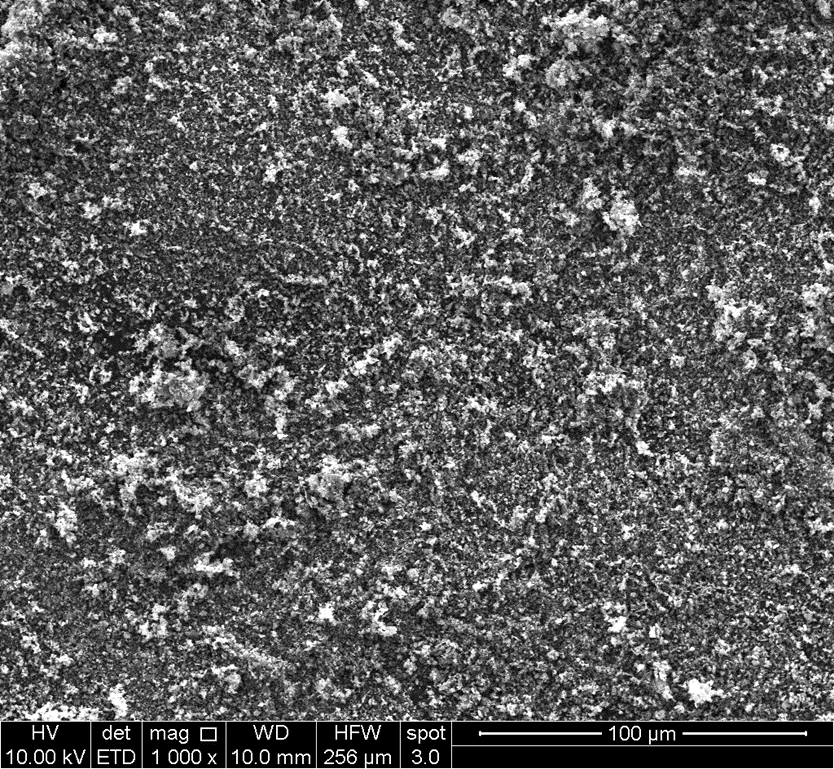 | 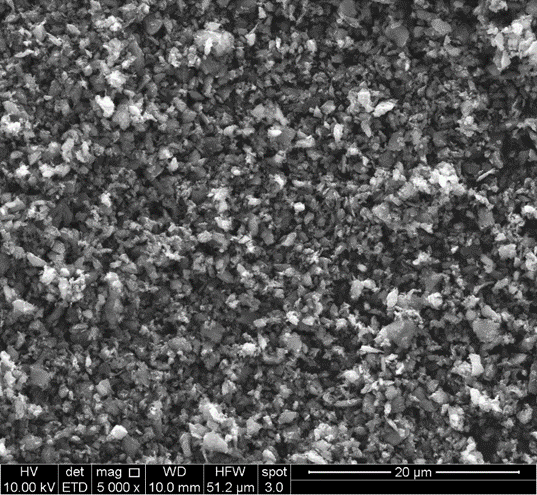 |
| --- | --- |

Figure S2. SEM images of grounded and ball-milled coal char

2. Characterization of carbon nanomaterials

Various characterizations are conducted on C1, C2 and C3. Lateral sizes and thicknesses are determined based on statistical TEM analysis. Some TEM and high resolution TEM images of C2 and C3 are shown in Fig. S3 and Fig. S4. The lateral sizes are summarized in Fig. S3 and Table S1. The elemental compositions are examined with XPS as shown in Table S2.

| 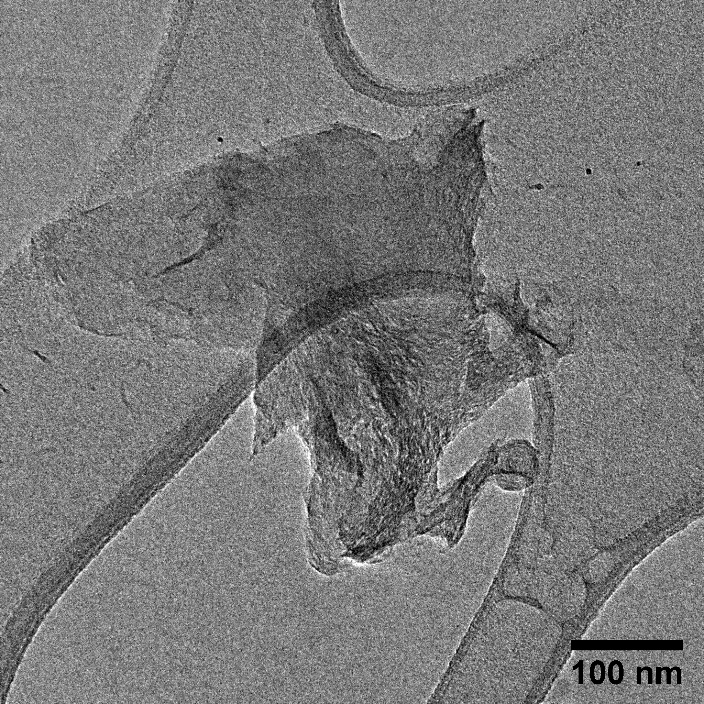 | 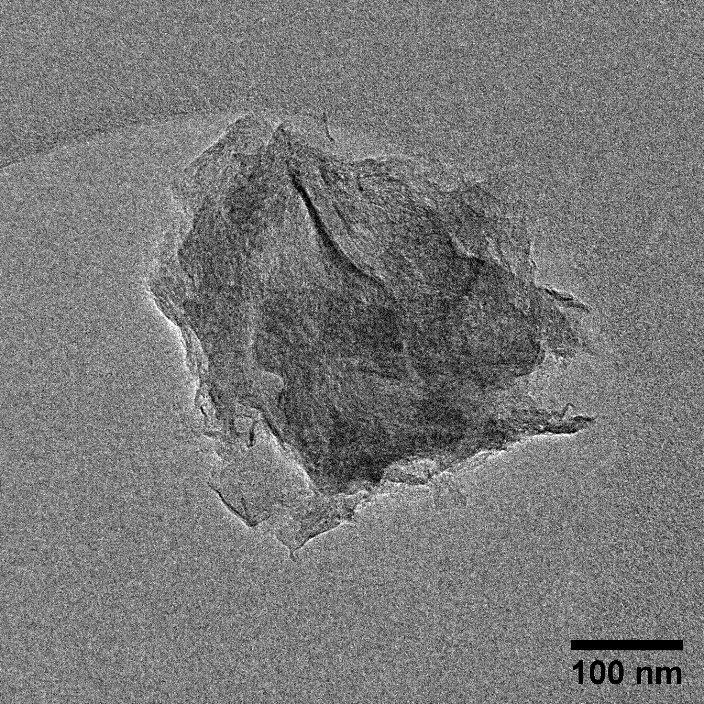 |
| --- | --- |
| 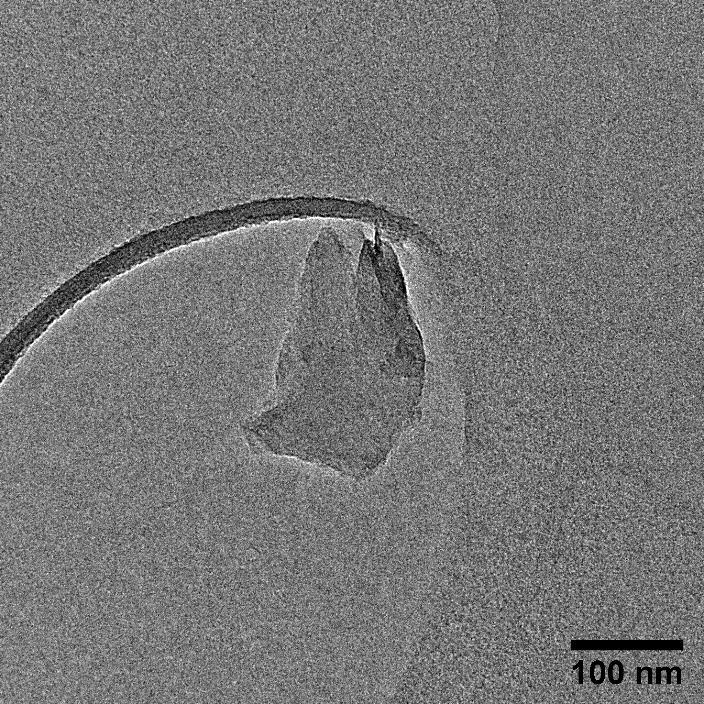 | 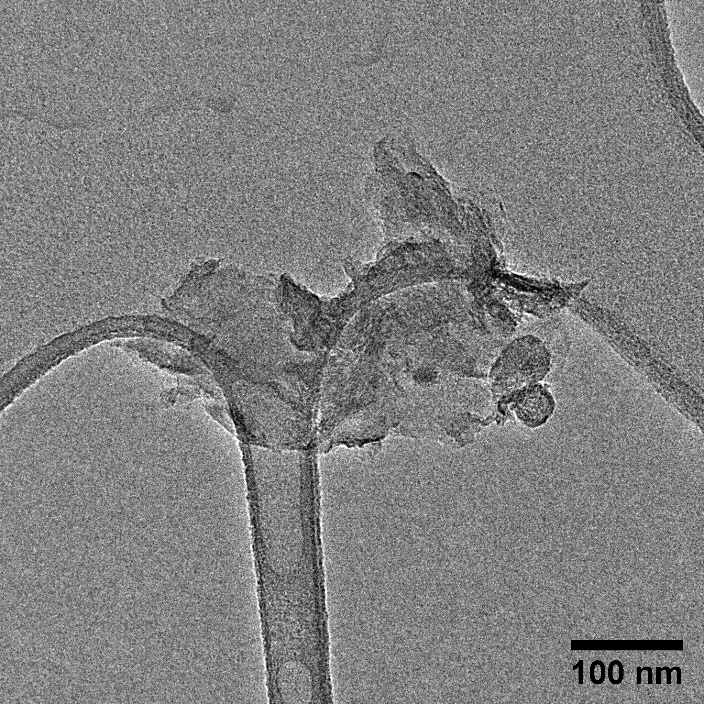 |
| 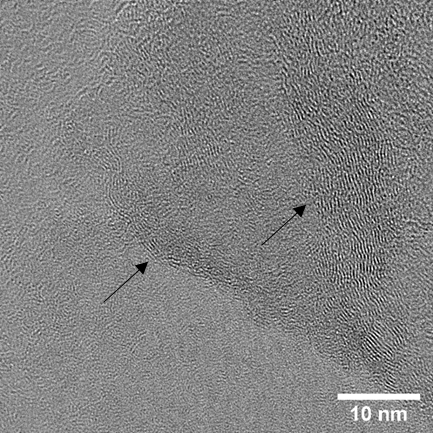 | 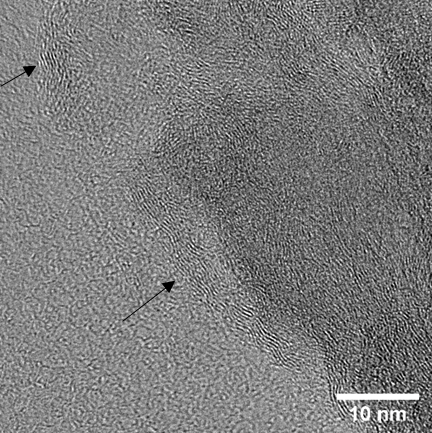 |
| 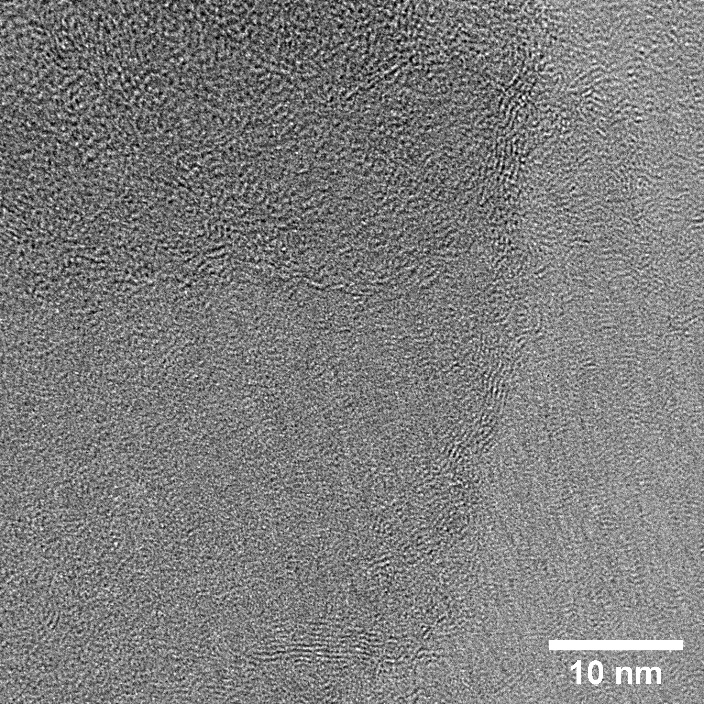 | 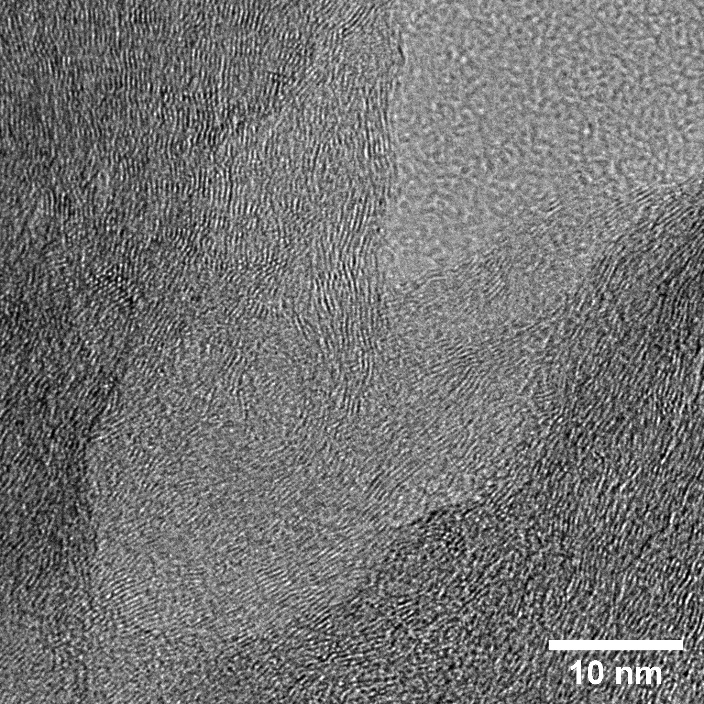 |
| 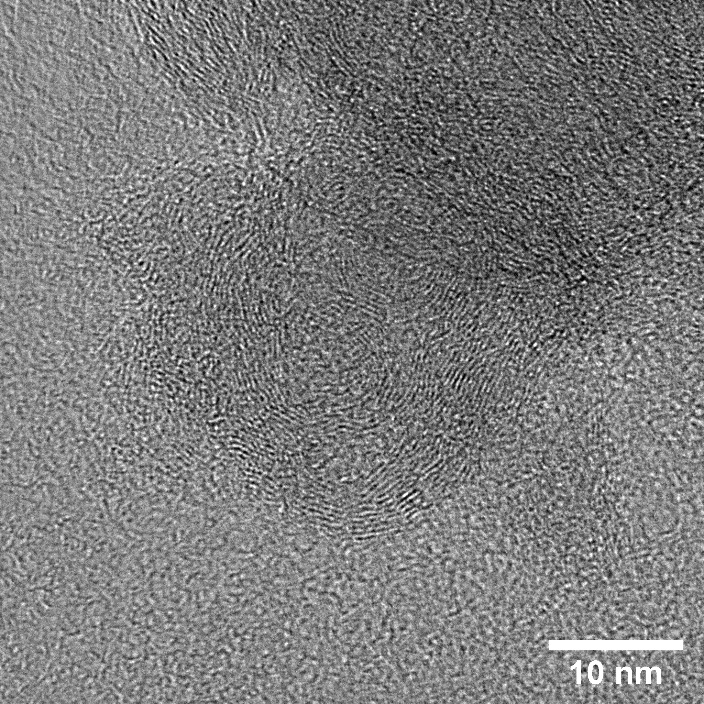 | 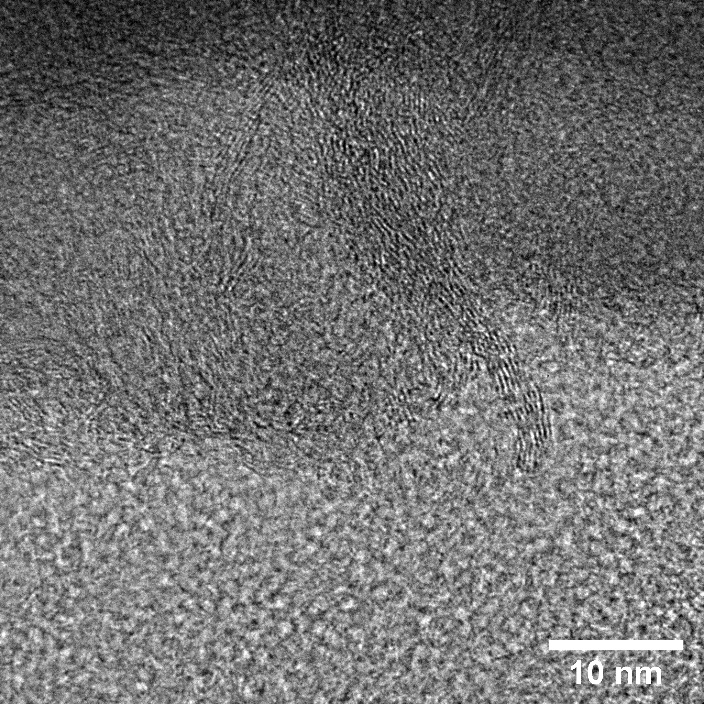 |

**Figure S3.** TEM images of C2

| 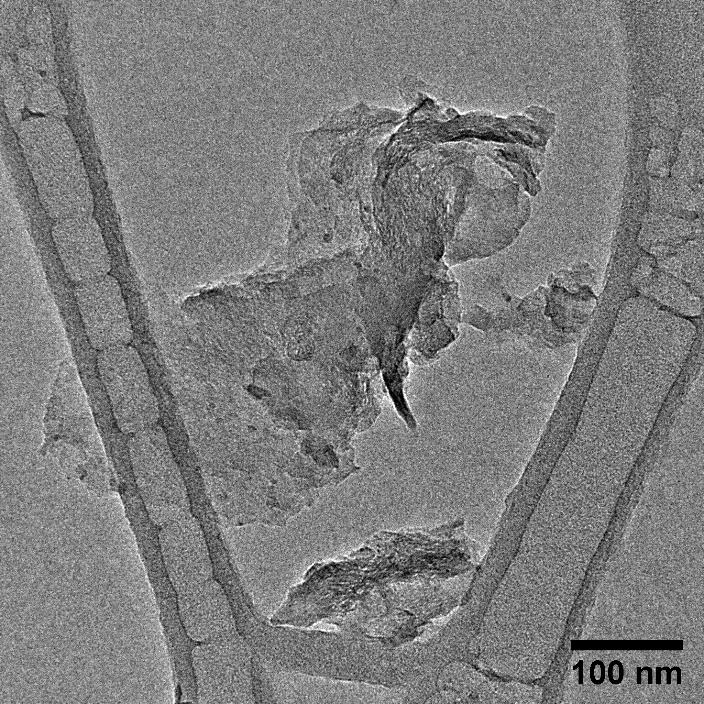 | 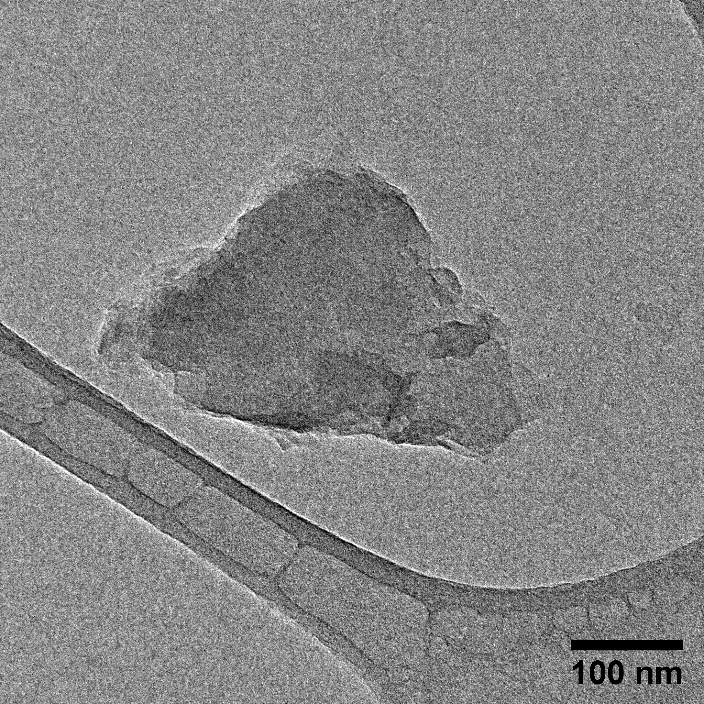 |
| --- | --- |
| 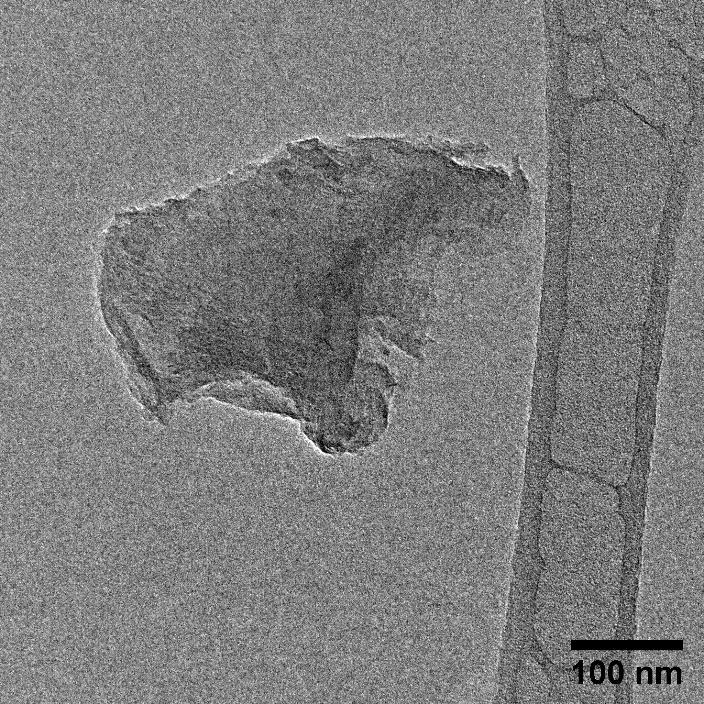 | 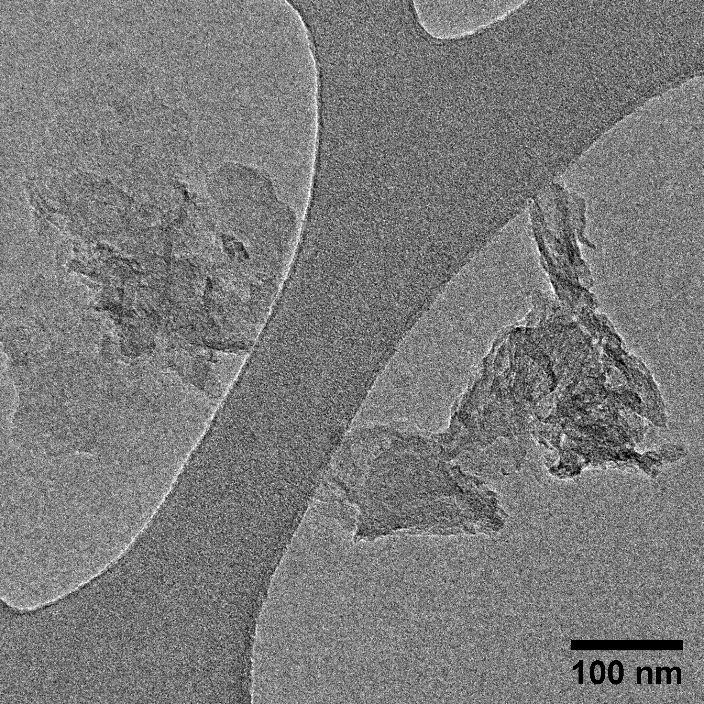 |
| 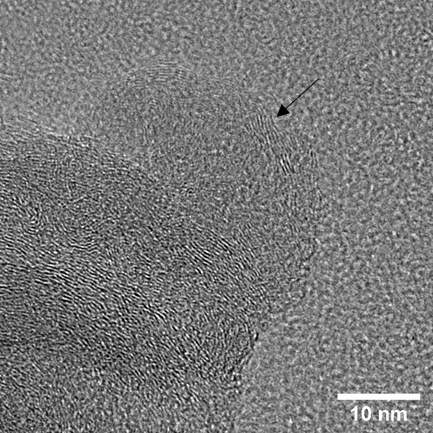 | 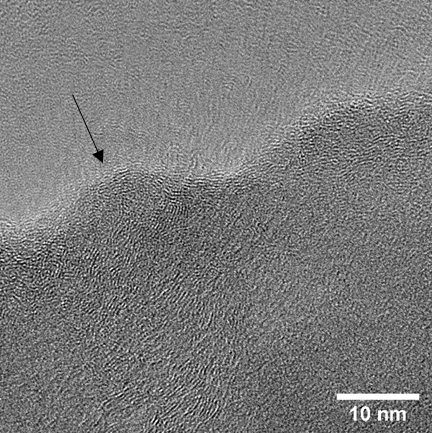 |
| 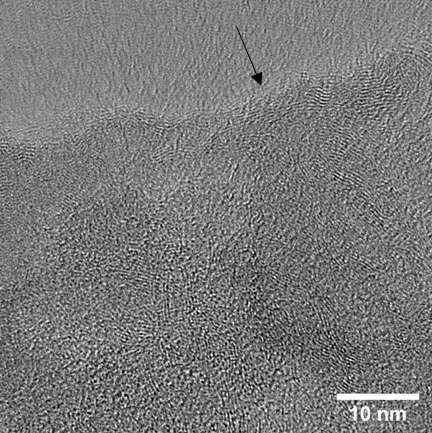 | 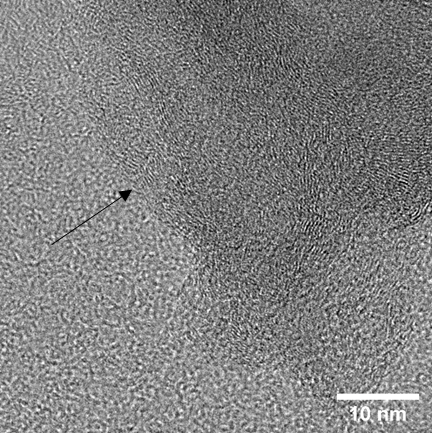 |
| 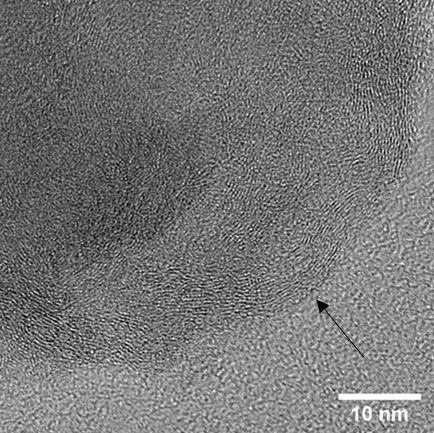 | 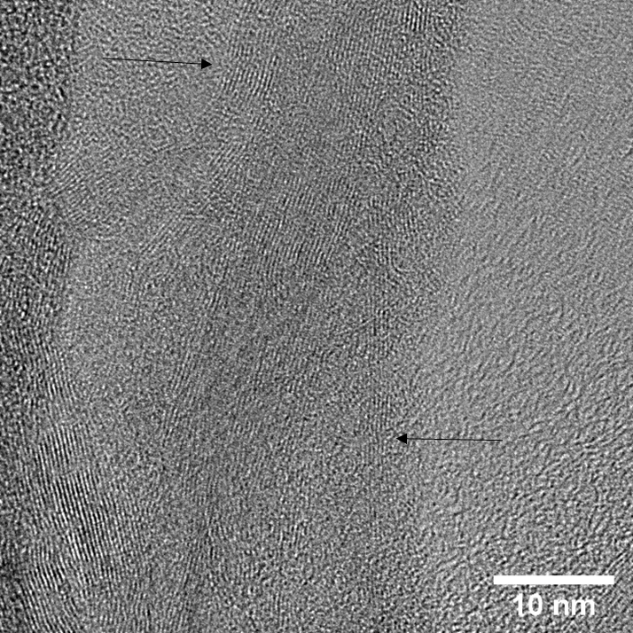 |

**Figure S4.** TEM images of C3

**Table S1**. Lateral sizes (nm) of C2 and C3

| Object number (arbitrary) | C2 | C3 |
| --- | --- | --- |
| 1 | 78 | 78 |
| 2 | 108 | 83 |
| 3 | 113 | 88 |
| 4 | 136 | 123 |
| 5 | 149 | 131 |
| 6 | 152 | 136 |
| 7 | 165 | 157 |
| 8 | 173 | 167 |
| 9 | 179 | 164 |
| 10 | 183 | 172 |
| 11 | 183 | 177 |
| 12 | 195 | 188 |
| 13 | 211 | 189 |
| 14 | 220 | 187 |
| 15 | 236 | 207 |
| 16 | 292 | 219 |
| 17 | 332 | 219 |
| 18 | 339 | 231 |
| 19 | 352 | 243 |
| 20 | 365 | 257 |
| 21 | 365 | 259 |
| 22 | 395 | 331 |
| 23 | 613 | 361 |
| 24 | 796 | 368 |
| 25 |  | 368 |
| 26 |  | 380 |
| 27 |  | 384 |
| 28 |  | 408 |
| 29 |  | 431 |
| Mean | 264 | 231 |
| STD | 162.5 | 102.8 |

**Table S2.** Elemental Composition of C1, C2, C3 and commercial GO

|  | **C1** | **C2** | **C3** | **GO**  **(from Graphenea)** |
| --- | --- | --- | --- | --- |
| **Elemental Composition**  **(atomic%)** | C-62.9%  O-35.0%  Fe-0.8%  Al-0.6%  Si-0.5%  S-0.3% | C-93.6%  O-5.0%  Al-0.9%  Si-0.2%  S-0.2%  Fe-0.1% | C-91.38%  O-6.82%  Al-0.47%  Ca-0.53%  S-0.37%  Si-0.22 | C-49-56%  O-41-50%  S-2-3%  H-0-1%  N-0-1% |

3. Agglomeration and dispersion of carbon nanomaterials within cement matrix

Similar to carbon nanotubes (CNTs) and other nanomaterials, good dispersion of graphene type materials is particularly difficult to achieve due to their high surface energy and strong Van der Waals interactions. In addition, the highly hydrophobic property of some graphene derivatives, in particular graphene nanoplatelets (GNPs), further complicates their dispersion in aqueous media. As a result, graphene sheets display a high tendency to form irreversible agglomerates or even to restack to form thick structures. Although graphene oxide (GO), compared to other forms of graphene materials, can be better dispersed in aqueous media due to electrostatic repulsion and hydrophilicity, its dispersion in cement composites has been found to be challenging as well. Due to the high concentration of alkaline ions (i.e. Ca^2+^. Na^+^, K^+^) in the solution of fresh cement pastes, the repulsion between GO nanosheets is weakened which leads to crosslinking of the GO sheets. Consequently, severe aggregation is often observed in experiments in both simulated pore solution[1] and cement paste[2], hampering the full utilization of GO for the mechanical improvement of cement-based materials. Coal-based carbon nanomaterials, C1, C2 and C3 have similar secondary agglomeration issue as graphene materials. Figure S4, shown below, illustrates the aggregation behavior of C1 that occurs in cement pore solutions due to presence of alkaline ions.


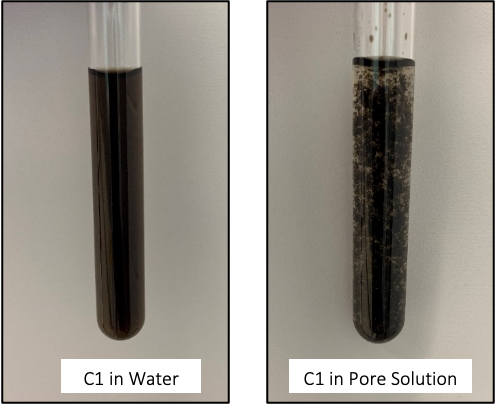


**Figure S4.** Secondary agglomeration of coal-derived C1 material in cement pore solution

Polycarboxylate-ether based superplasticizer (SP), widely used as a water-reducing agent in concrete industry, has been a popular dispersing agent for carbon nanomaterials in cement matrix. The SP is able to cover the surface of the carbon nano-additive in case of Ca^2+^ adsorption, and lower the free Ca^2+^ concentration due to the strong complexation which impedes the cross-linking of Ca^2+^ through steric hindrance effect. The ratio between SP and the carbon nanomaterials has to be carefully determined, where excessive SP could reduce the workability of fresh cement and impact the mechanical properties after the cement hardens. Fig. S5 shows the effect of SP on delaying the agglomeration of carbon nanomaterials within the cement pore solution.


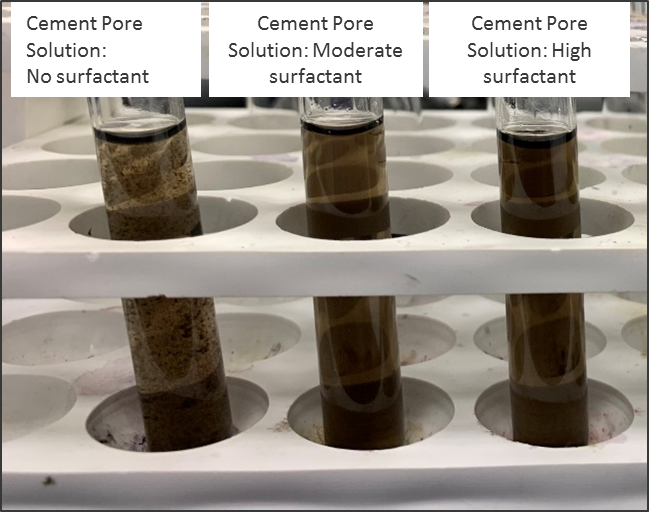


**Figure S5.** Effect of SP on preventing the agglomeration of C1 in cement pore solution.

4. List of reported mechanical properties improvement of Nanomaterials enhanced cement composite (GNP: graphene nanoplatelet, GO: graphene oxide)

**Table S3.** Literature reported mechanical property reinforcement with different graphene materials synthesized from graphite as well as with other nanomaterials (at 28 days age)

| Filler | Ref# | Cement Composite | Optimum Dosage | Compressive Enhancement | Flexural Enhancement |
| --- | --- | --- | --- | --- | --- |
| GNP | [3] | Paste | 0.06 wt% | 11% | 27.8% |
| GNP | [4] | Paste | 0.05 wt% | 24% | 8% |
| GNP | [5] | Mortar | 0.05 wt% | 8.3% | 15.6% |
| GNP | [6] | Concrete | 0.05 wt% | 22.4% | -- |
| GNP | [7] | Concrete | 0.025 wt% | 17% | 6% |
| GNP | [8] | UHPC | 0.5 wt% | 43.5% | 39.1% |
| GO | [9] | Paste | 0.025 wt% | 37% | 66% |
| GO | [10] | Paste | 0.02 wt% | 29% | -- |
| GO | [11] | Paste | 0.1 wt% | 26% | 21% |
| GO | [12] | Mortar | 0.01-0.03 wt% | 5.16% | 21.86% |
| GO | [13] | Mortar | 0.022 wt% | 22.6% | 24.6% |
| GO | [14] | Concrete | 0.08 wt% | 21% | 21% |
| GO | [15] | UHPC | 0.02 wt% | 25.3% | 28.7% |
| Nanoclay | [16] | Concrete | 10 wt% | 63% | 47% |

**References**

[1] L. Zhao, X. Guo, Y. Liu, C. Ge, Z. Chen, L. Guo, X. Shu, J. Liu, Investigation of dispersion behavior of GO modified by different water reducing agents in cement pore solution, Carbon. 127 (2018) 255–269. https://doi.org/10.1016/j.carbon.2017.11.016.

[2] X. Li, A.H. Korayem, C. Li, Y. Liu, H. He, J.G. Sanjayan, W.H. Duan, Incorporation of graphene oxide and silica fume into cement paste: A study of dispersion and compressive strength, Construction and Building Materials. 123 (2016) 327–335. https://doi.org/10.1016/j.conbuildmat.2016.07.022.

[3] W. Baomin, D. Shuang, Effect and mechanism of graphene nanoplatelets on hydration reaction, mechanical properties and microstructure of cement composites, Construction and Building Materials. 228 (2019) 116720. https://doi.org/10.1016/j.conbuildmat.2019.116720.

[4] B. Wang, R. Jiang, Z. Wu, Investigation of the Mechanical Properties and Microstructure of Graphene Nanoplatelet-Cement Composite, Nanomaterials (Basel, Switzerland). 6 (2016) 200. https://doi.org/10.3390/nano6110200.

[5] J. Tao, X. Wang, Z. Wang, Q. Zeng, Graphene nanoplatelets as an effective additive to tune the microstructures and piezoresistive properties of cement-based composites, Construction and Building Materials. 209 (2019) 665–678. https://doi.org/10.1016/j.conbuildmat.2019.03.173.

[6] G. Chen, M. Yang, L. Xu, Y. Zhang, Y. Wang, Graphene nanoplatelets impact on concrete in improving freeze-thaw resistance, Applied Sciences. 9 (2019) 3582.

[7] Z. Jiang, O. Sevim, O.E. Ozbulut, Mechanical properties of graphene nanoplatelets-reinforced concrete prepared with different dispersion techniques, Construction and Building Materials. 303 (2021) 124472. https://doi.org/10.1016/j.conbuildmat.2021.124472.

[8] S. Dong, Y. Wang, A. Ashour, B. Han, J. Ou, Nano/micro-structures and mechanical properties of ultra-high performance concrete incorporating graphene with different lateral sizes, Compos. Part A Appl. Sci. Manuf. 137 (2020) 106011. https://doi.org/10.1016/j.compositesa.2020.106011.

[9] M. Birenboim, R. Nadiv, A. Alatawna, M. Buzaglo, G. Schahar, J. Lee, G. Kim, A. Peled, O.Regev, Reinforcement and workability aspects of graphene-oxide-reinforced cement nanocomposites, Composites Part B: Engineering. 161 (2019) 68–76. https://doi.org/10.1016/j.compositesb.2018.10.030.

[10] G. Xu, S. Du, J. He, X. Shi, The role of admixed graphene oxide in a cement hydration system, Carbon. 148 (2019) 141–150. https://doi.org/10.1016/j.carbon.2019.03.072.

[11] W.-J. Long, J.-J. Wei, F. Xing, K.H. Khayat, Enhanced dynamic mechanical properties of cement paste modified with graphene oxide nanosheets and its reinforcing mechanism, Cement and Concrete Composites. 93 (2018) 127–139. https://doi.org/10.1016/j.cemconcomp.2018.07.001.

[12] H. Peng, Y. Ge, C.S. Cai, Y. Zhang, Z. Liu, Mechanical properties and microstructure of graphene oxide cement-based composites, Construction and Building Materials. 194 (2019) 102–109. https://doi.org/10.1016/j.conbuildmat.2018.10.234.

[13] L. Zhao, X. Guo, C. Ge, Q. Li, L. Guo, X. Shu, J. Liu, Mechanical behavior and toughening mechanism of polycarboxylate superplasticizer modified graphene oxide reinforced cement composites, Composites Part B: Engineering. 113 (2017) 308–316. https://doi.org/10.1016/j.compositesb.2017.01.056.

[14] S.C. Devi, R.A. Khan, Effect of graphene oxide on mechanical and durability performance of concrete, Journal of Building Engineering. 27 (2020) 101007. https://doi.org/10.1016/j.jobe.2019.101007.

[15] Y.-Y. Wu, J. Zhang, C. Liu, Z. Zheng, P. Lambert, Effect of Graphene Oxide Nanosheets on Physical Properties of Ultra-High-Performance Concrete with High Volume Supplementary Cementitious Materials., Mater. (Basel, Switzerland). 13 (2020). https://doi.org/10.3390/ma13081929.

[16] A. Mansi, N.H. Sor, N. Hilal, S. M A Qaidi, The impact of Nano Clay on Normal and High-Performance Concrete Characteristics: A Review., IOP Conf. Ser.: Earth Environ. Sci. 961 (2022). https://doi.org/10.1088/1755-1315/961/1/012085
